# Multimodal sensory information is represented by a combinatorial code in a sensorimotor system

**DOI:** 10.1371/journal.pbio.2004527

**Published:** 2018-10-15

**Authors:** Rosangela Follmann, Christopher John Goldsmith, Wolfgang Stein

**Affiliations:** School of Biological Sciences, Illinois State University, Normal, Illinois, United States of America; EPFL, Switzerland

## Abstract

A ubiquitous feature of the nervous system is the processing of simultaneously arriving sensory inputs from different modalities. Yet, because of the difficulties of monitoring large populations of neurons with the single resolution required to determine their sensory responses, the cellular mechanisms of how populations of neurons encode different sensory modalities often remain enigmatic. We studied multimodal information encoding in a small sensorimotor system of the crustacean stomatogastric nervous system that drives rhythmic motor activity for the processing of food. This system is experimentally advantageous, as it produces a fictive behavioral output in vitro, and distinct sensory modalities can be selectively activated. It has the additional advantage that all sensory information is routed through a hub ganglion, the commissural ganglion, a structure with fewer than 220 neurons. Using optical imaging of a population of commissural neurons to track each individual neuron's response across sensory modalities, we provide evidence that multimodal information is encoded via a combinatorial code of recruited neurons. By selectively stimulating chemosensory and mechanosensory inputs that are functionally important for processing of food, we find that these two modalities were processed in a distributed network comprising the majority of commissural neurons imaged. In a total of 12 commissural ganglia, we show that 98% of all imaged neurons were involved in sensory processing, with the two modalities being processed by a highly overlapping set of neurons. Of these, 80% were multimodal, 18% were unimodal, and only 2% of the neurons did not respond to either modality. Differences between modalities were represented by the identities of the neurons participating in each sensory condition and by differences in response sign (excitation versus inhibition), with 46% changing their responses in the other modality. Consistent with the hypothesis that the commissural network encodes different sensory conditions in the combination of activated neurons, a new combination of excitation and inhibition was found when both pathways were activated simultaneously. The responses to this bimodal condition were distinct from either unimodal condition, and for 30% of the neurons, they were not predictive from the individual unimodal responses. Thus, in a sensorimotor network, different sensory modalities are encoded using a combinatorial code of neurons that are activated or inhibited. This provides motor networks with the ability to differentially respond to categorically different sensory conditions and may serve as a model to understand higher-level processing of multimodal information.

## Author summary

Nervous systems are continuously challenged by the need of processing stimuli from various sensory modalities that may arrive simultaneously or in disparate order. How these stimuli are encoded and separated so that organisms can carry out appropriate behavioral responses is an ongoing topic of high interest. We studied this question using a ganglion with fewer than 220 neurons in the crab nervous system. The neurons in this ganglion process mechanosensory and chemosensory information to control aspects of feeding. Using fluorescence imaging to track the activity of individual neurons, we provide evidence that differences between modalities are encoded in the combination of activated neurons. Specifically, while 80% of the imaged neurons responded to both modalities, differences between modalities were represented by the sign of the response, i.e., whether neurons were excited or inhibited. Moreover, we found a new combination of excitation and inhibition when both pathways were activated simultaneously. These data are consistent with the idea that this small sensorimotor network encodes different sensory modalities in a combinatorial code of neurons. Each perceived modality results in a different combination of activated and inhibited neurons, providing the downstream motor networks with the ability to differentially respond to distinct categories of sensory conditions.

## Introduction

Integrating information from multiple sensory modalities and producing appropriate motor outputs are vital functions of the nervous system, and the neural networks underlying these two functions are tightly linked in both vertebrates and invertebrates. From a traditional perspective, individual senses are first integrated separately and subsequently combined at numerous multimodal convergence zones, including cortical and subcortical regions [1], as well as multimodal association areas [2–4]. More recent observations of multimodal responses in once-considered modality-specific regions, however, suggest that multimodal processing is a ubiquitous function of the nervous system rather than a localized feature [5].

With respect to motor control, multimodal interactions are thought to mainly occur in upstream motor control circuits [6], despite some evidence for multimodal convergence in downstream sensorimotor circuits that directly initiate and modulate behavioral actions [7–9]. Some examples include the vertebrate brain stem and spinal cord [10,11] but also arthropod thoracic and commissural ganglia (CoGs) [12–14]. Although responses to individual sensory modalities and their consequences for motor output in these downstream sensorimotor networks are often well characterized, little is known about the mechanisms by which multimodal information is encoded. Examples of premotor multisensory integration come from single-neuron studies in the superior colliculus [8,15–18], showing that multiple sensory modalities are processed in a distributed fashion throughout this brain stem region, with some neurons being exclusively unimodal and other being multimodal. Hypotheses of encoding of multimodal information include changes in neuronal firing rates (e.g., a rate code) [19], activation of distinct network components, or distinct activation and inhibition of neurons within a shared population (e.g., a combinatorial code) [20,21].

Central pattern generators and their premotor networks comprise a type of sensorimotor circuit that is particularly amenable to studying multimodal information processing. While central pattern generators can produce stereotypic rhythmic activity in the absence of sensory input, their activity patterns vary in different sensory and modulatory conditions [22–25]. They are controlled by descending projection neurons that innervate the pattern generators and adjust motor output to different behavioral conditions [9,26,27]. These descending neurons are well-characterized building blocks of sensorimotor processing in both vertebrates and invertebrates and relay sensory information processed by local networks to the central pattern generators [28–36]. The link between converging sensory pathways and motor control in these networks provides a means to investigate how multimodal sensory information is represented in the immediate context of behavioral output and may provide a model for more complex multimodal brain areas.

It is well established that sensory pathways that innervate these control networks activate the descending projection neurons and that this has functional consequences for downstream motor output, like switching from ingestion to egestion in *Aplysia* feeding [37] and forward-to-backward walking in *Drosophila* [34]. Nonetheless, with the exception of a few well-characterized projection neurons, it is unknown how sensory information is integrated as a whole in the networks the projection neurons are embedded in. Moreover, it is unclear how these networks respond to and encode multimodal information. In the crustacean stomatogastric nervous system, a small pool of descending projection neurons resides within the paired CoGs, each of which contain fewer than 220 neurons [38]. They integrate information from different sensory modalities [39–41]. We used these advantageous features to investigate the coding mechanism for multiple sensory modalities presented both individually (unimodal inputs) and simultaneously (bimodal input). Specifically, we selectively activated the chemosensory inferior ventricular neurons (IVs) and the mechanosensory ventral cardiac neurons (VCNs) with physiologically relevant stimulation parameters [42,43]. Each sensory modality causes a distinct response in a small set of identified descending projection neurons [40,41,44] via fast monosynaptic inputs but also elicits slower and more sustained responses through polysynaptic actions [40,44] that likely stem from local interneurons in the CoGs. The resulting projection neuron activity then alters motor output of the downstream stomatogastric motor circuits [45], providing a direct link between sensory pathways and motor activity.

Using multineuron imaging with a voltage-sensitive fluorescent dye, we study how the local CoG network encodes sensory information from several modalities. We show that 98% of the local CoG neurons are involved in processing of chemosensory and mechanosensory information, with 80% of the neurons being multimodal. Differences between modalities were represented by which neurons responded to a particular pathway—i.e., the identities of the neurons participating—and by differences in response sign—i.e., whether neurons were excited or inhibited by sensory stimulation. The pyloric rhythm showed different activity patterns in the two sensory conditions, and these differences depended on CoG input. Bimodal input was encoded by a set of neurons distinct from either unimodal condition. This suggests that the CoG network employs a combinatorial code to represent different sensory modalities, enabling the downstream motor circuits to differentially respond to these conditions.

## Results

### A network of commissural neurons processes information from multiple sensory modalities

**Fig 1:**
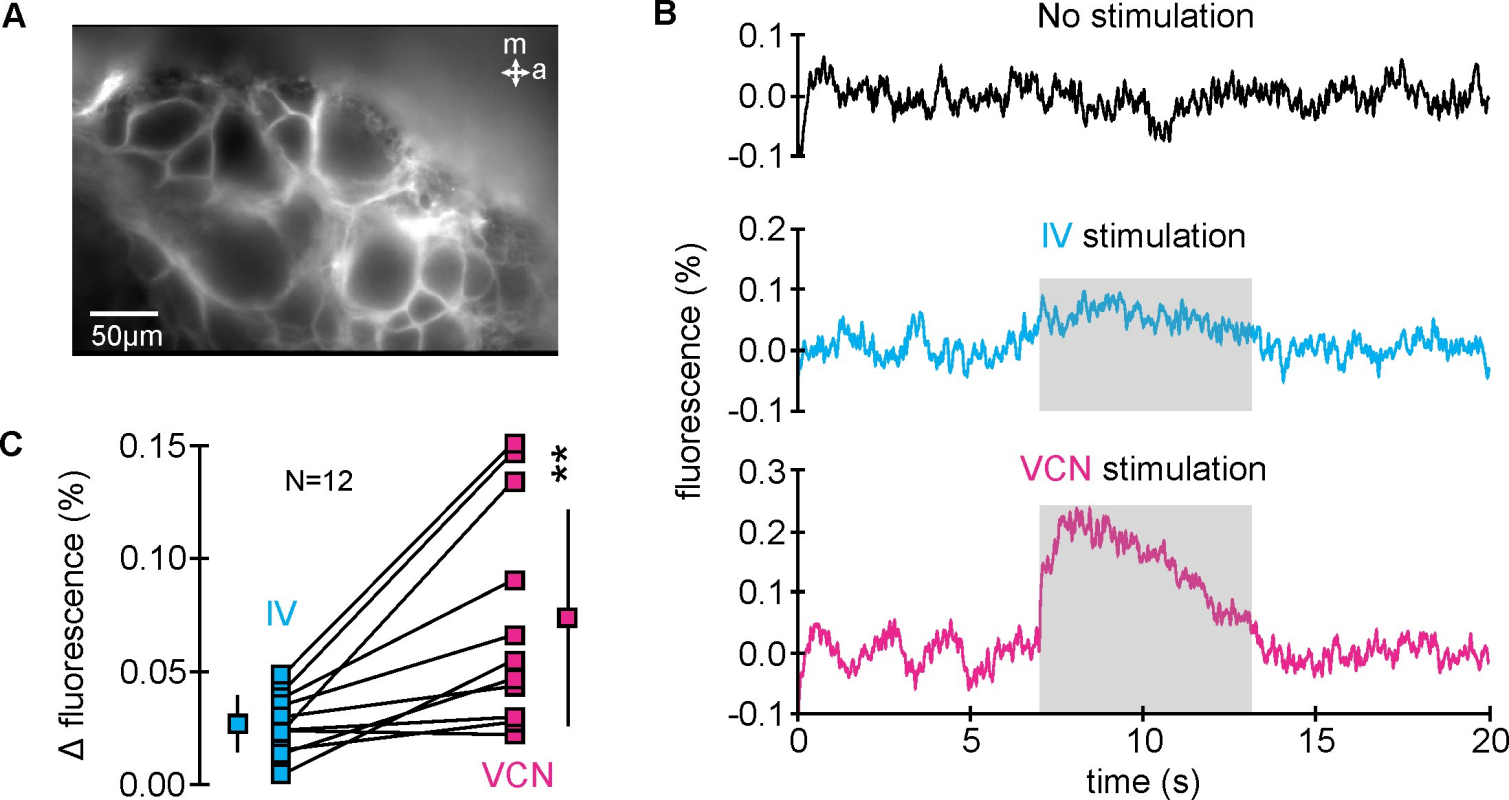
Multiple sensory modalities converge in the CoG and elicit distinct neuronal responses. (A) Fluorescence image of the CoG area that contains the cell bodies of the neurons involved in control of downstream stomatogastric motor circuits [38]. (B) Averaged fluorescence signals from all neurons without stimulation (top), during IV stimulation (middle), and during VCN stimulation (bottom). IV and VCN stimulation both caused an increase in fluorescence. Stimulus trains lasted for 6 seconds (shaded region). (C) Plot of the average changes in fluorescence between prestimulation control and stimulation (Δ fluorescence) for both stimulus conditions. The magnitude of the CoG response was significantly larger during VCN stimulation (*N* = 12, **p < 0.01, paired *t* test, the underlying data can be found in S1 Data). Data from individual ganglia and their mean ± SD are provided. CoG, commissural ganglion; IV, inferior ventricular neuron; VCN, ventral cardiac neuron.

The chemosensory (IV) and mechanosensory (VCN) pathways are known to modulate downstream motor patterns, and these effects are relayed through CoG neurons [40,41,44]. Previous studies demonstrated that these two pathways converge onto the same four identified descending premotor neurons, eliciting distinct responses and providing preliminary evidence for the presence of a combinatorial code to encode multimodal information [39,40]. The projection neurons are also part of a larger network of CoG neurons, since their sensory responses are at least partly due to polysynaptic inputs and in response to longer-lasting and continuous stimuli [40,43]. For example, while some projection neurons receive monosynaptic postsynaptic potentials from the VCN, they also show more complex and longer-lasting synaptic potentials that include polysynaptic inhibitory components. There is also evidence suggesting that projection neurons release neurotransmitter in the CoGs and thus act as local interneurons ([46]; Nusbaum, personal communication). It is unclear how extensive the local CoG network is and how sensory information from multiple different modalities is represented in it. To characterize the sensory response of the CoG premotor region, we employed multineuron imaging with fast voltage-sensitive dyes while selectively stimulating IV and VCN pathways. A key advantage of this technique is the combination of single-cell imaging and high temporal resolution [47]. We focused on the medial-posterior region of the CoG, a region that also contains the cell bodies of the projection neurons innervating the downstream stomatogastric ganglion (STG) [38] (Fig 1A and S1 Fig, dashed rectangles). We first averaged the optical signals of all cell bodies in this area to test whether other neurons in this regions are involved in sensory processing at all. In addition, this allowed an initial assessment of the CoG network response (Fig 1B). Both IV and VCN modalities induced changes in fluorescence over the course of the stimulus train (IV = 40 Hz stimulation frequency; VCN = 15 Hz stimulation frequency; Fig 1B), reflecting the change of activity within the CoG network in response to each sensory stimulation. The magnitude of the VCN-induced response was consistently greater than that elicited by IV stimulation (*N* = 12, *p* < 0.05, paired *t* test; Fig 1C), suggesting that IV and VCN modalities are processed differently by the CoG network.

**Fig 2:**
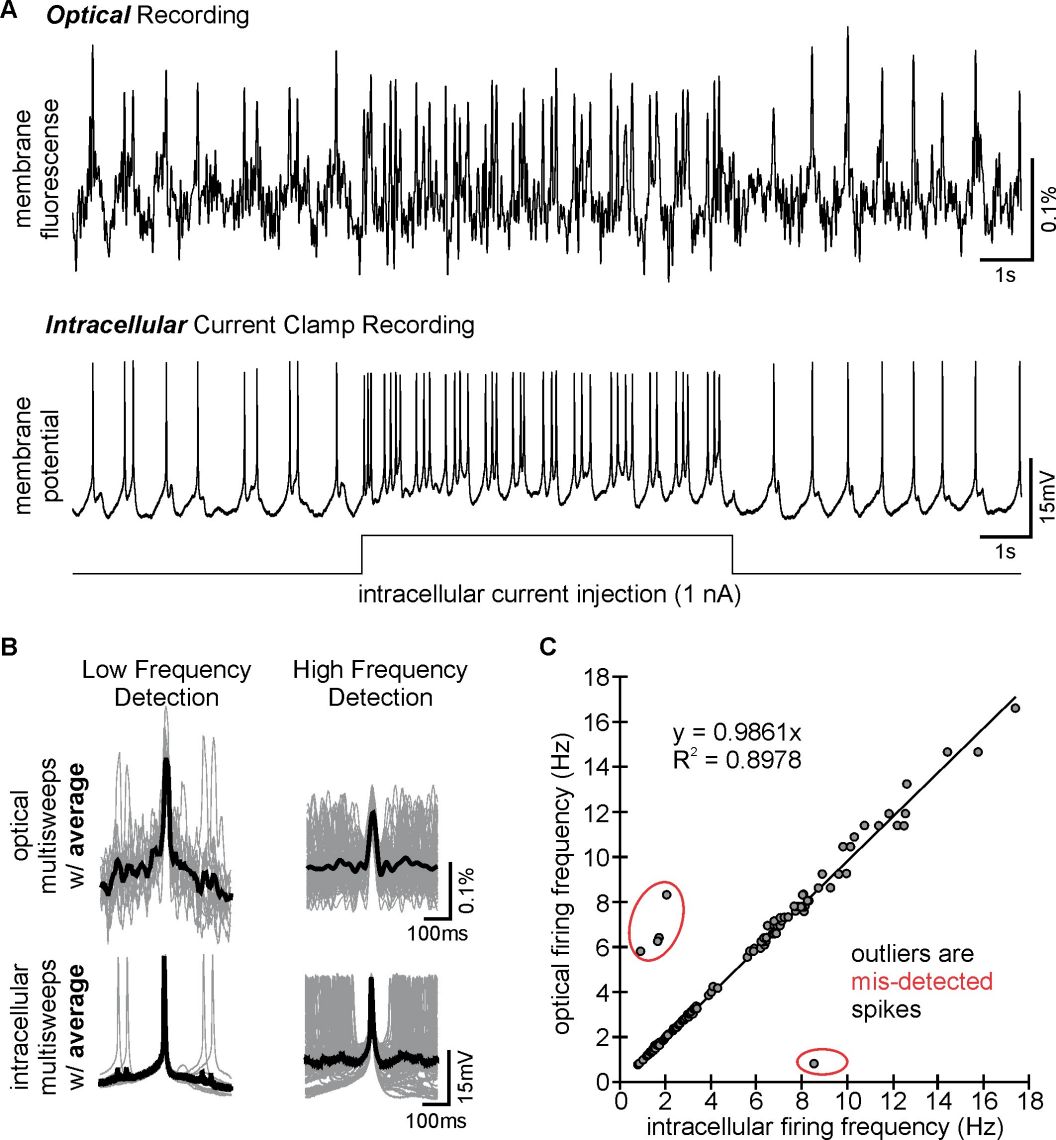
VSD imaging reliably detects CoG neuron firing. Comparison of optical recording with VSDs with an intracellular recording acquired simultaneously in the same CoG neuron. (A) Single-sweep measurements from a representative optical (top) and intracellular (bottom) recording, demonstrating accuracy of optical spike detection during both spontaneous low-frequency firing (approximately 1 Hz) and induced high-frequency firing (15–20 Hz). High frequencies were induced with depolarizing current injection through the microelectrode. (B) Optical (top) and intracellular (bottom) multisweeps (gray overlaid traces) for low and high spike frequencies. Waveform averages (black thick trace) are overlaid on the multisweep traces. (C) Using spike-to-spike latency measurements, we compared the efficiency of the optical recording in detecting spikes. Outliers are misdetected action potentials and circled in red and are indicative of either overdetection or underdetection in the optical recording. CoG, commissural ganglion; VSD, voltage-sensitive dye.

To test whether these differences were indicative of a distinct recruitment of neuronal populations or rather of different neuronal response intensities within the same population (or combinations thereof), we analyzed neuronal spike activities with single-cell resolution. Optical spike detection is reliably possible in CoG neurons. Fig 2A compares the activity of a single CoG neuron using simultaneous intracellular and optical recordings. Action potentials were reliably detected in both recordings, with the optical recording having a sufficiently high signal-to-noise ratio to correctly detect action potentials (similar to previous recordings in this system, [48]). Only 6 out of the 114 action potentials from the shown neuron were misdetected in the optical recording. Fig 2B shows a comparison of spike-triggered overlays of optical and intracellular waveforms, plus averages. As reported previously [48], action potentials are easily detected, although having a lower signal-to-noise ratio than those recorded with intracellular electrodes. This was also the case in our recordings and likely a consequence of undersampling in the optical recording (sample rate = 250 frames/second). The optical recording also allowed assessment of slower subthreshold events, although in most of our subsequent analyses, we focused on action potential firing frequencies. Fig 2C shows the high correlation between optically and electrically detected action potential frequencies.

**Fig 3:**
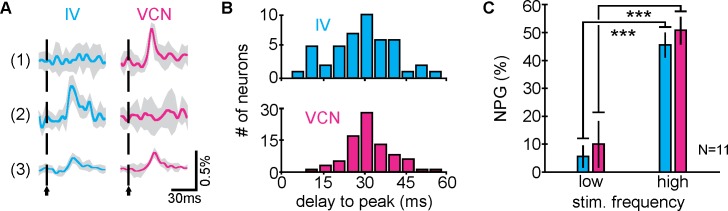
Neuronal response latencies are similar between sensory modalities. (A) Responses of three individual neurons (1–3) to IV (left) and VCN (right) low-frequency stimuli (1 Hz) in the same preparation. Vertical dashed lines indicate stimulus onset. Data are averaged traces of six trials ± SD (shaded region). (B) Distribution of response latencies (time from stimulus onset to peak neuronal response) for neurons excited by IV (top) and VCN (bottom) low-frequency stimuli (1 Hz). There were no significant differences between distributions (*N* = 11 ganglia, *n* = 129 neurons, *p* = 0.140, two-sample Kolmogorov-Smirnov test). Mean delays, IV: 29.7 ± 11.4 ms, VCN: 31.9 ± 7.7 ms. (C) Excitatory responses to low-frequency (1 Hz) and high-frequency (IV: 40 Hz; VCN: 15 Hz) stimuli. High frequencies excited more neurons in both sensory conditions (the underlying data can be found in S1 Data). IV, inferior ventricular neuron; NPG (%), number of neurons per ganglion, in percent; VCN ventral cardiac neuron.

To assess how sensory stimuli are represented in the CoG network, we probed single-neuron activity in different sensory conditions. Initially, we aimed to address the question how many neurons are involved in the processing and whether sensory information is processed locally in the CoG network or only relayed through it. We used a low-frequency (1 Hz) stimulation protocol to test for short-latency connections that should mainly activate neurons directly influenced by the sensory pathways. We found neurons that were reliably excited during only IV or VCN stimulation and some neurons that were excited by both modalities (stimulated at different times, examples shown in Fig 3A). There were no sensory-specific differences in mean CoG neuron response latency (Fig 3B, *N* = 11, *p* = 0.19, unpaired two-sample *t* test) and no difference in the distribution of latencies across all CoG neurons (*N* = 11, *n* = 129 neurons, *p* = 0.14, two-sample Kolmogorov-Smirnov [K-S] test; Fig 3B). However, on average, very few neurons were excited. During IV stimulation, only 5.7% ± 3.8% (*N* = 11) were excited versus 10.3% ± 8.2% during VCN stimulation (*N* = 11, Fig 3C). When we used physiologically relevant stimulation frequencies (i.e., stimulation frequencies known to elicit long-lasting gastric mill activity in stomatogastric motor neurons; a 6-second train of either 40 Hz [IV] or 15 Hz [VCN] [42,43]), significantly more neurons were excited by either stimulation (IV: 45.7% ± 4.3%; VCN: 50.9% ± 4.8%; *N* = 11, *p* < 0.001, paired *t* test; Fig 3C), suggesting that physiologically relevant stimuli sum up substantially, or act via polysynaptic interactions and involve a larger number of local neurons.

**Fig 4:**
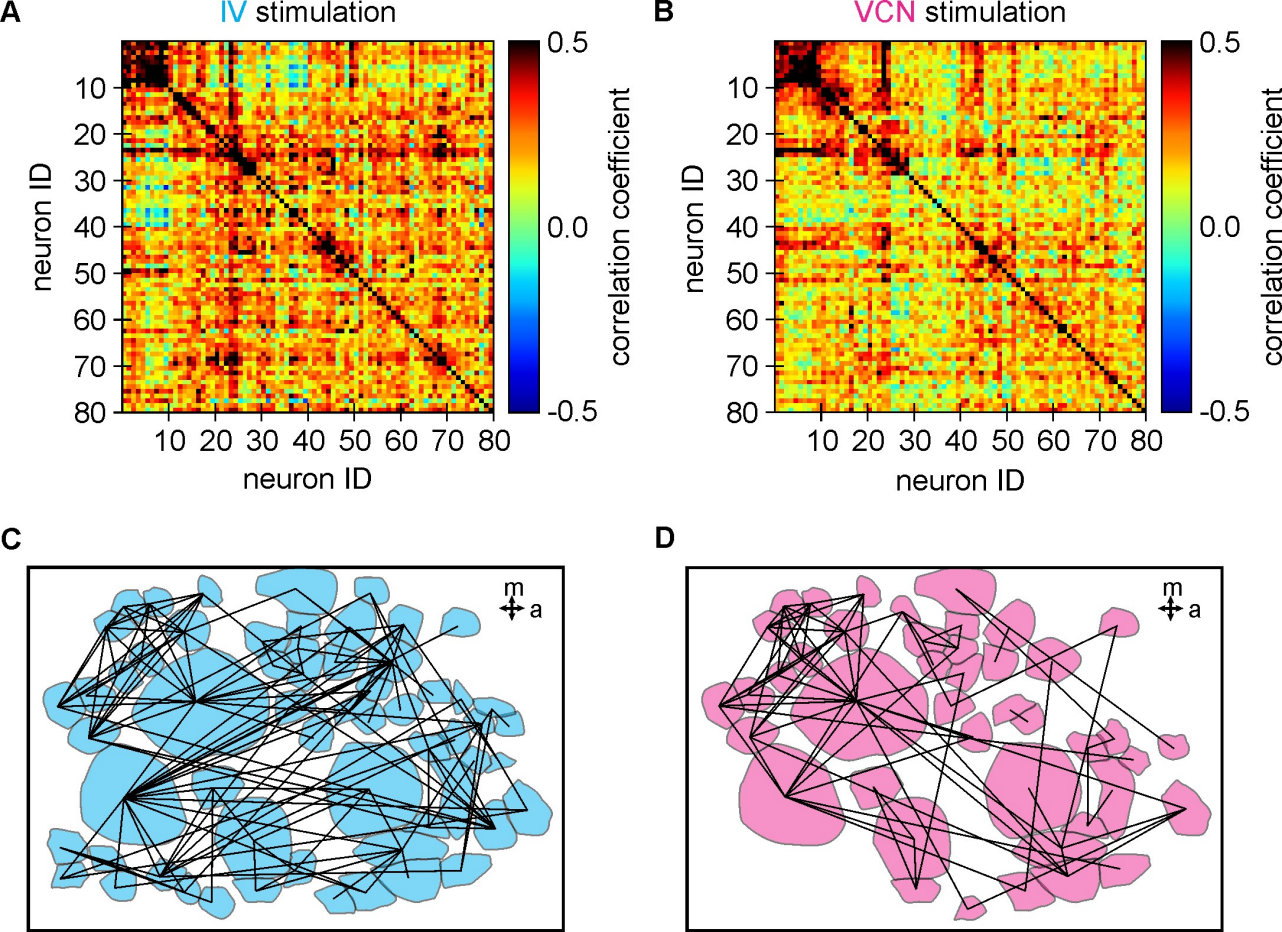
Functional connectivity maps revealed a dense and efficient network for information transfer. (A) Coherence color map during IV stimulation for one preparation. (B) Coherence color map during VCN stimulation for one preparation. Darker colors indicate higher coherence between pairs of neurons. The coherence matrix had a threshold of 0.45. Pairs of neurons with a correlation coefficient above this threshold were considered functionally connected. (C) Functional connectivity map during IV stimulation. (D) Functional connectivity map during VCN stimulation for the same preparation. IV, inferior ventricular neuron; VCN ventral cardiac neuron.

Indeed, most imaged neurons (>98%) (*N* = 12 ganglia, *n* = 878 neurons) responded in some manner to sensory stimulation, and less than 2% were unaffected by either stimulation. To further assess whether the responding neurons acted as a coherent group to integrate sensory stimuli or if sensory stimuli were processed in a segregated manner, we constructed functional connectivity maps for each preparation in the two sensory conditions (Fig 4, see Materials and methods). In short, we established the functional connectivity by calculating the Pearson correlation between the activity traces of all pairs of neurons in a given preparation, leading to a coherence matrix for each condition. Fig 4 shows coherence color maps during IV (Fig 4A) and VCN (Fig 4B) stimulation for an individual ganglion and the corresponding functional connectivity maps (Fig 4C and 4D). We used two parameters commonly used to describe complex networks—namely, connectivity density and global efficiency—to characterize the functional network structures in the two sensory conditions. Connectivity density measures how well connected a network is. If all potential connections existed, connectivity density would have a value of 1. Biological neuronal networks, however, employ only a small fraction of the potential connections. In the example of Fig 4, 80 cells were imaged, which resulted in a functional network with 134 connections during IV stimulation and 81 connections during VCN stimulation with a connectivity density of 4.2% (IV condition) and 2.5% (VCN condition). Taking all experiments together, the connectivity density for IV was 7.43% ± 4.1% and 5.34% ± 3.2% for VCN (*N* = 19, 14 crabs), which, on average, is larger than that reported for *Caenorhabditis elegans* (3.85%) [49,50] but smaller than cortex [51].

The global efficiency parameter provides a measure of the network’s capacity for parallel information transfer between neurons. It is a function of the inverse of the shortest path length between each pair of nodes [52] and gives a measure of how efficiently neurons communicate information. In *C*. *elegans*, for instance, the nervous system has a global efficiency of 0.46 [53]. In the CoG examples shown, the efficiency was 0.21 for the IV condition and 0.08 for the VCN condition. On average, the global efficiency was 0.25 ± 0.1 for the IV condition and 0.18 ± 0.09 for the VCN condition (*N* = 19 ganglia, 14 crabs). These data indicate that within a given modality, there is considerable parallel processing of sensory information by the responding CoG neurons, with relatively dense functional connections between neurons. Together, these data suggest that for each modality, the CoG neurons formed a coherent functional network that integrates sensory information from both modalities.

### Similarities in CoG spike frequency distributions between modalities contradict a network rate code

**Fig 5:**
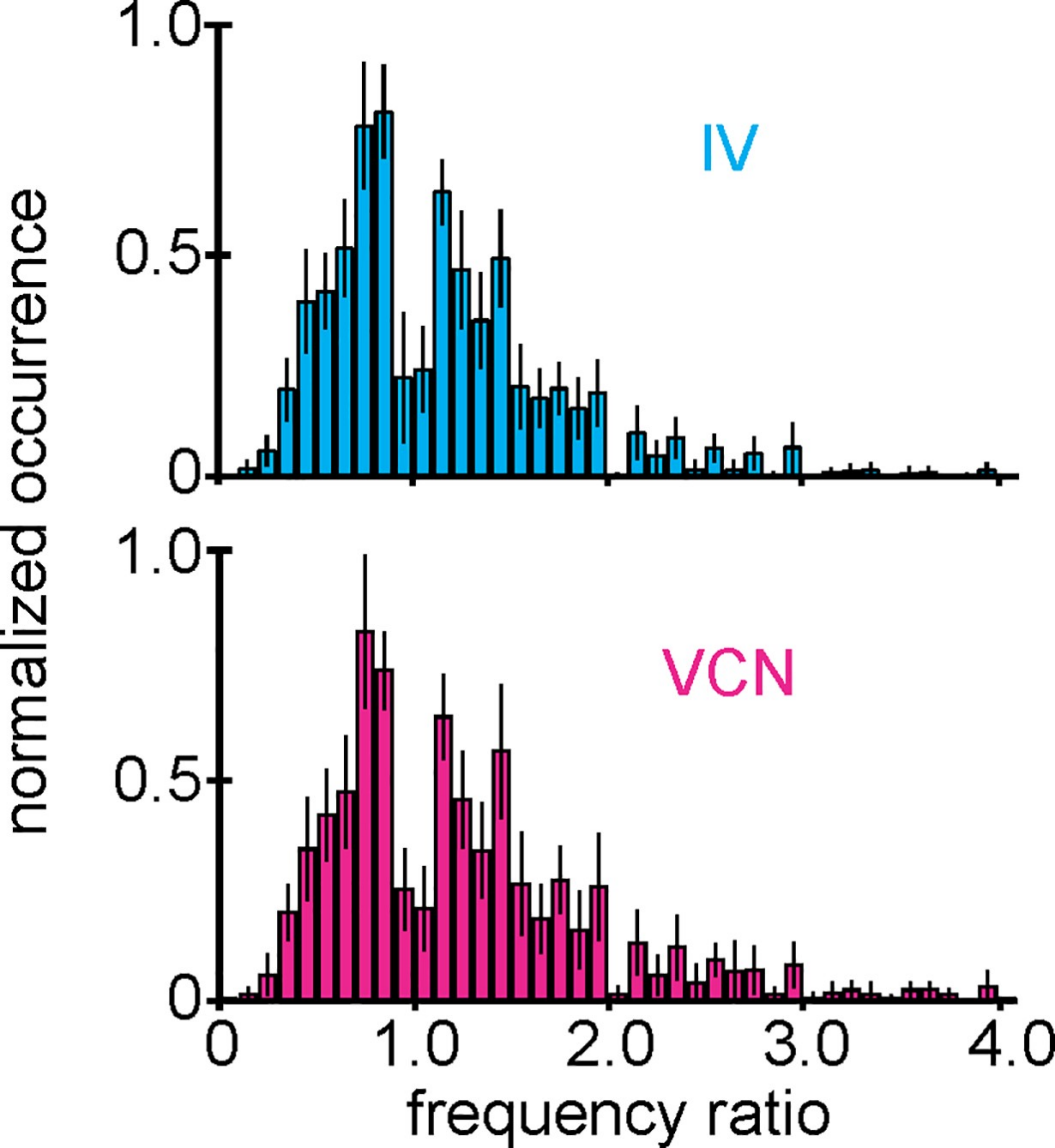
The distribution of CoG neuron firing responses is similar between sensory modalities. High-frequency sensory stimuli yielded various neuronal responses that altered spontaneous activity levels. Spike frequency ratio distributions of all neurons for IV (top) and VCN (bottom) inputs. No differences were found (*N* = 12 ganglia, 9 crabs, *n* = 878 neurons, *p* = 0.144, two-sample Kolmogorov-Smirnov test, S1 Data). Data are mean ± SD. CoG, commissural ganglion; IV, inferior ventricular neuron; VCN, ventral cardiac neuron.

How does the CoG network encode the different sensory modalities? One possibility would be to encode differences between modalities in the firing frequencies of the CoG neurons. For individual modalities, there is evidence that firing frequencies change with the strength of the sensory input and that these changes determine the response of the downstream motor pattern [54]. Although unlikely to exist as a means to separate different modalities, there are also indications that CoG neuron firing rates can differ between modalities [40]. A rate-coding mechanism predicts that the same CoG neurons are recruited by IV and VCN stimulation, with the distinguishing information between modalities being encoded in the firing frequency responses of these neurons. If this was the case, the CoG network distribution of firing responses should thus differ between modalities. Across all conditions, neurons, and ganglia, CoG neuron firing frequencies ranged from 0.33 to 14 Hz (*N* = 12). Most CoG neurons were spontaneously active even in the absence of sensory stimuli, at rates of 0.33–6.8 Hz (N = 12). When we compared the range of firing frequencies associated with IV and VCN conditions, we found no differences between them: 0.33–14.00 Hz (IV) and 0.33–13.80 Hz (VCN). More importantly, there was no difference in the distribution of firing frequency changes induced by each condition. Fig 5 shows the distribution of normalized frequency changes for both pathways, using the ratio of CoG neuron firing frequency (frequency ratio) during stimulation (*stim*_*ON*_) to the frequency preceding stimulus presentation (*stim*_*PRE*_, compare S1 Fig). Over the entire network (*N* = 12, *n* = 878 neurons), there was no difference in this distribution (*N* = 12, *p* = 0.144, K-S test; Fig 5), indicating that no distinct firing frequencies and thus no network rate code existed for distinguishing between sensory modalities. Per definition, however, when considering the ratio, neurons that were not spontaneously active had to be omitted. Thus, we also tested the distributions of the frequency differences between sensory stimulation and spontaneous activity immediately before stimulation. We found no differences in the frequency difference distribution either (*N* = 12, 9 crabs, *n* = 878, *p* = 0.5, K-S test).

### Distinct neuron responses support a combinatorial code for multimodal representation

**Fig 6:**
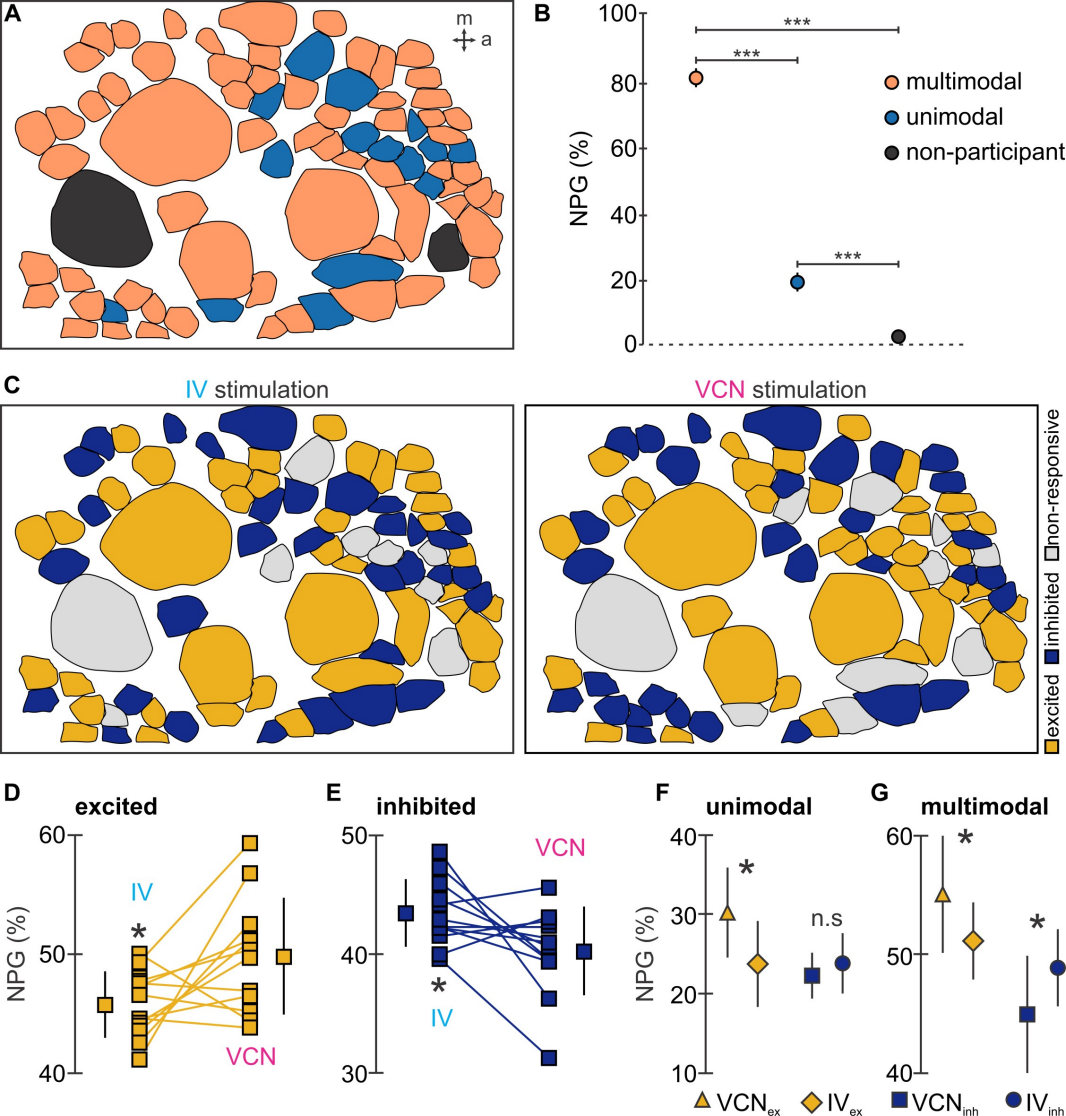
Network excitation and inhibition is modality specific. (A) Example map showing multimodal (neurons that responded to both sensory modalities, orange cell bodies), unimodal (neurons that responded to only one sensory modality, blue cell bodies), and nonparticipant (neurons that did not respond to either modality, black cell bodies) CoG neurons. (B) Across ganglia, a significantly greater proportion of multimodal neurons was found in comparison to the proportion unimodal neurons, and only a small fraction of neurons were nonparticipants. The plot shows the normalized NPG. Data are mean ± SD, (***p < 0.001, repeated measures ANOVA with Tukey-Kramer post hoc test). (C) Example response maps illustrating the types of neuronal responses induced by stimulation of IV (left) and VCN (right) pathways: excited (yellow), inhibited (blue), or nonresponsive (gray). (D) Proportion of NPG that were excited by IV and VCN stimulation, as indicated. VCN stimulation excited a significantly larger proportion of CoG neurons than IV stimulation. (E) Proportion of NPG that were inhibited by IV and VCN stimulation, as indicated. IV stimulation inhibited a significantly larger number of CoG neurons than VCN stimulation. (F) Normalized NPG of unimodal response types. The largest proportion of unimodal neurons were excited by VCN stimulation. (G) Normalized NPG of multimodal response types. Mirroring the results in Fig 6D and Fig 6E, a larger proportion of (in this case, multimodal) CoG neurons were excited by VCN stimulation, and a larger proportion were inhibited by IV stimulation. Data (D, E, F, and G) are mean ± SD (*N* = 12 ganglia for each response type compared, 9 crabs, *p < 0.05, paired *t* test, the underlying data can be found in S1 Data). CoG, commissural ganglion; IV, inferior ventricular neuron; NPG, number of neurons per ganglion; n.s., not significant; VCN, ventral cardiac neuron.

The large number of participating neurons supports the idea that most of the imaged population contributed to sensory encoding but also suggested overlap of the circuits processing each input instead of having distinct pathways for each modality. To differentiate between these possibilities, we mapped each individual ganglion and classified neurons as multimodal if they responded to both sensory stimulations, unimodal if they responded to only one, or nonresponsive. Fig 6A shows an example spatial map of unimodal and multimodal neurons from one CoG. Indeed, the majority of neurons (about 80%) were multimodal responders; i.e., they responded (with excitation or inhibition) to both IV and VCN stimulation. About 18% of the neurons were unimodal responders, responding to only one of the two pathways, and 2% did not respond (Fig 6B; *N* = 12, F[2,22] = 5,075.48, *p* < 0.001, repeated measures ANOVA, Tukey-Kramer post hoc test). Moreover, the variability associated with these proportions was low across preparations; i.e., there was a consistent subset of neurons that were specific to only one or the other modality despite the high degree of overlap in the CoG neurons responding to each modality. It is worth noting, though, that the number of multimodal neurons is likely an underestimate, because we only tested two sensory modalities, while several other modalities are known to influence CoG neurons. It is thus likely that neurons not responding at all may respond to additional sensory stimuli that were not tested by our approach. Following the same argument, the 18% of neurons that only responded to one or the other modality may respond to additional modalities. These findings clearly contradicted the idea of separate pathways for each modality. They are, however, consistent with a combinatorial code, since many neurons responded to both sensory modalities but showed distinct responses to them.

In addition, if a combinatorial code was present, one would expect that individual neurons in the multimodal group show distinct responses in the two sensory conditions. To address this, we assessed the proportions of excited and inhibited neurons in both modalities, i.e., whether neurons that are either excited or inhibited by one modality showed the opposite response for the other modality. We generated activity maps of neurons that were excited, inhibited, or “nonresponsive” (no change in firing frequency) to IV (Fig 6C, left) and VCN (Fig 6C, right) stimulation. Neuronal responses were distinct between modalities: more neurons per ganglion were excited by VCN stimulation than by IV stimulation (VCN = 49.8% ± 4.8%, IV = 45.7% ± 2.7%, *N* = 12, *p* < 0.05 paired *t* test; Fig 6D), supporting our initial finding that VCN stimulation yields a higher proportion of excitation in CoG neurons (Fig 1C). Conversely, IV stimulation yielded a larger number of inhibited neurons per ganglion than VCN stimulation (IV = 43.8% ± 3.0%, VCN = 40.3% ± 3.9%, *N* = 12, *p* < 0.05 paired *t* test; Fig 6E). These categorical differences were maintained when we split the analysis into unimodal and multimodal groups: significantly more unimodal and multimodal neurons were excited by VCN than by IV input (Fig 6F and 6G; *N* = 12, *p* < 0.05 each, paired *t* test). IV stimulation caused inhibition of significantly more multimodal neurons than VCN stimulation (IV_inh_ = 48.9% ± 3.2%; VCN_inh_ = 44.9% ± 4.9%, *N* = 12, *p* < 0.05, paired *t* test), accounting for the observed differences in modality-specific inhibition in Fig 6E.

More importantly, a large proportion of the multimodal neurons changed their response from excitatory to inhibitory or vice versa. In total, 46.15% ± 1.9% (*N* = 12 ganglia) multimodal neurons changed their response, further providing support that differences between modalities are encoded in distinct combinations of neurons.

**Fig 7:**
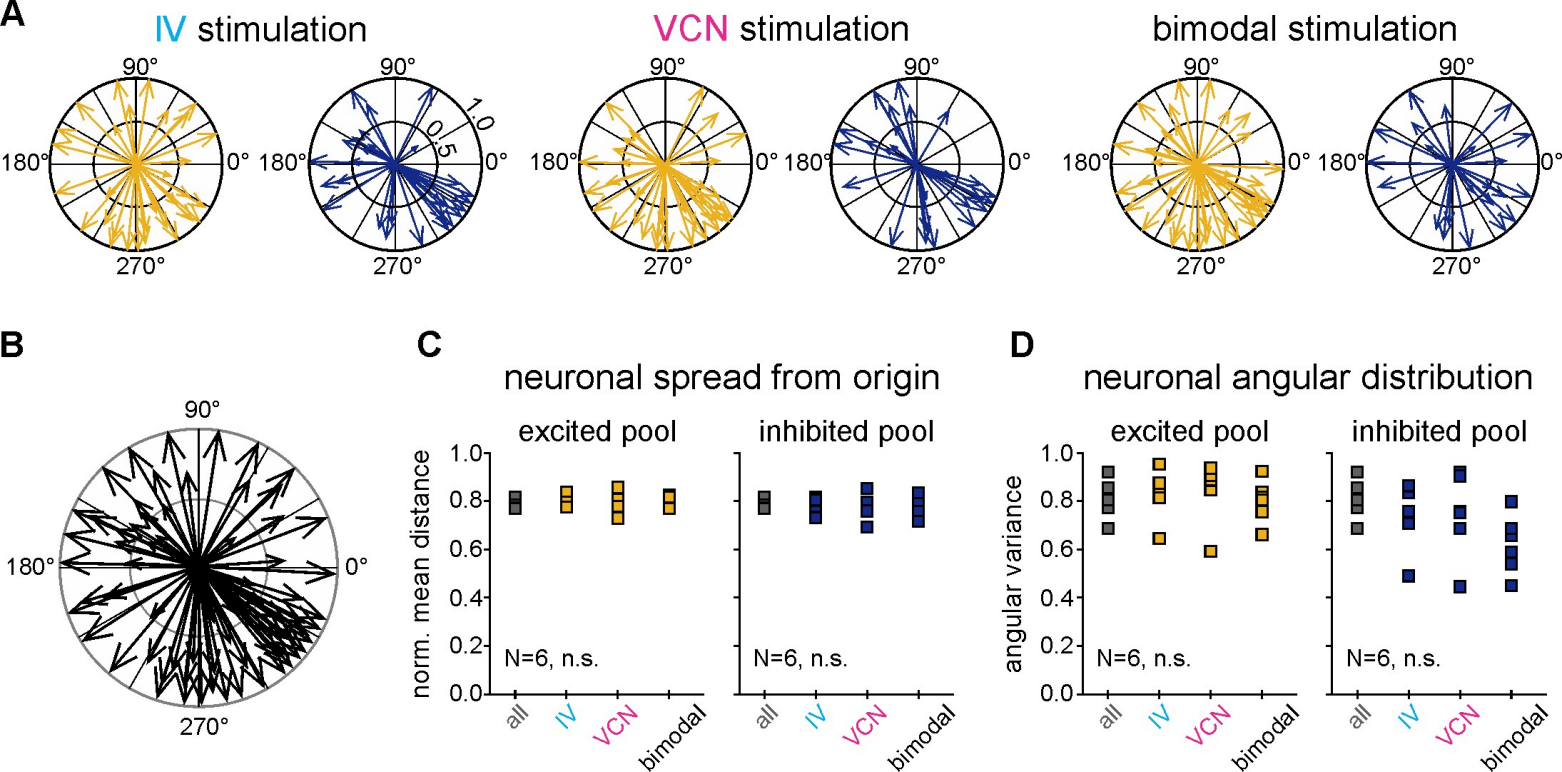
No differences in the spatial distributions of CoG neurons were found between different sensory conditions or neuronal response types. (A) Vector plot of excited and inhibited neurons for (from left to right) IV, VCN, and bimodal stimulation showing the orientation vectors with respect to the center of the imaged region for one ganglion (see Materials and methods). Vector lengths correspond to the normalized distance from the center. (B) Vector plot of all imaged cell bodies from the example used in (A). (C) Normalized mean distance from the origin for each condition (*N* = 6 ganglia, *p* = 0.24 for excited neurons, *p* = 0.87 for inhibited neurons, repeated measures ANOVA, Tukey-Kramer post hoc test). (D) Angular variance for each sensory condition (*N* = 6 ganglia, 4 crabs *p* = 0.88 for excited neurons, *p* = 0.11 for inhibited neurons, repeated measures ANOVA, Tukey-Kramer post hoc test). No differences in distance and angular distributions were found across sensory condition and response types (the underlying data can be found in S1 Data). CoG, commissural ganglion; IV, inferior ventricular neuron; n.s., not significant; VCN, ventral cardiac neuron.

The generated activity maps also allowed us to test whether neurons responding to a particular sensory modality were spatially clustered, a feature often observed in larger brain areas [55–57]. To determine if such a spatial clustering existed in the CoGs, we calculated the directional vector and distance from the center of the imaged area for each imaged neuron. Fig 7A shows the obtained vectors for a given ganglion for each sensory condition, separated for excited and inhibited neurons. Fig 7B shows them combined for all conditions. Across animals, there was no significant difference in the distance from the center for any condition or type of response (Fig 7C, *N* = 6 ganglia, *p* = 0.88 for excited neurons, *p* = 0.11 for inhibited neurons, repeated measures ANOVA, Tukey-Kramer post hoc test). Similarly, there was no difference between directional vectors for any condition and type of neuron (Fig 7D, *N* = 6 ganglia, *p* = 0.24 for excited neurons, *p* = 0.87 for inhibited neurons, repeated measures ANOVA, Tukey-Kramer post hoc test). Thus, sensory processing was distributed throughout the whole CoG network, and no specific areas were responsive to one modality or showed specific excitatory or inhibitory responses.

**Fig 8:**
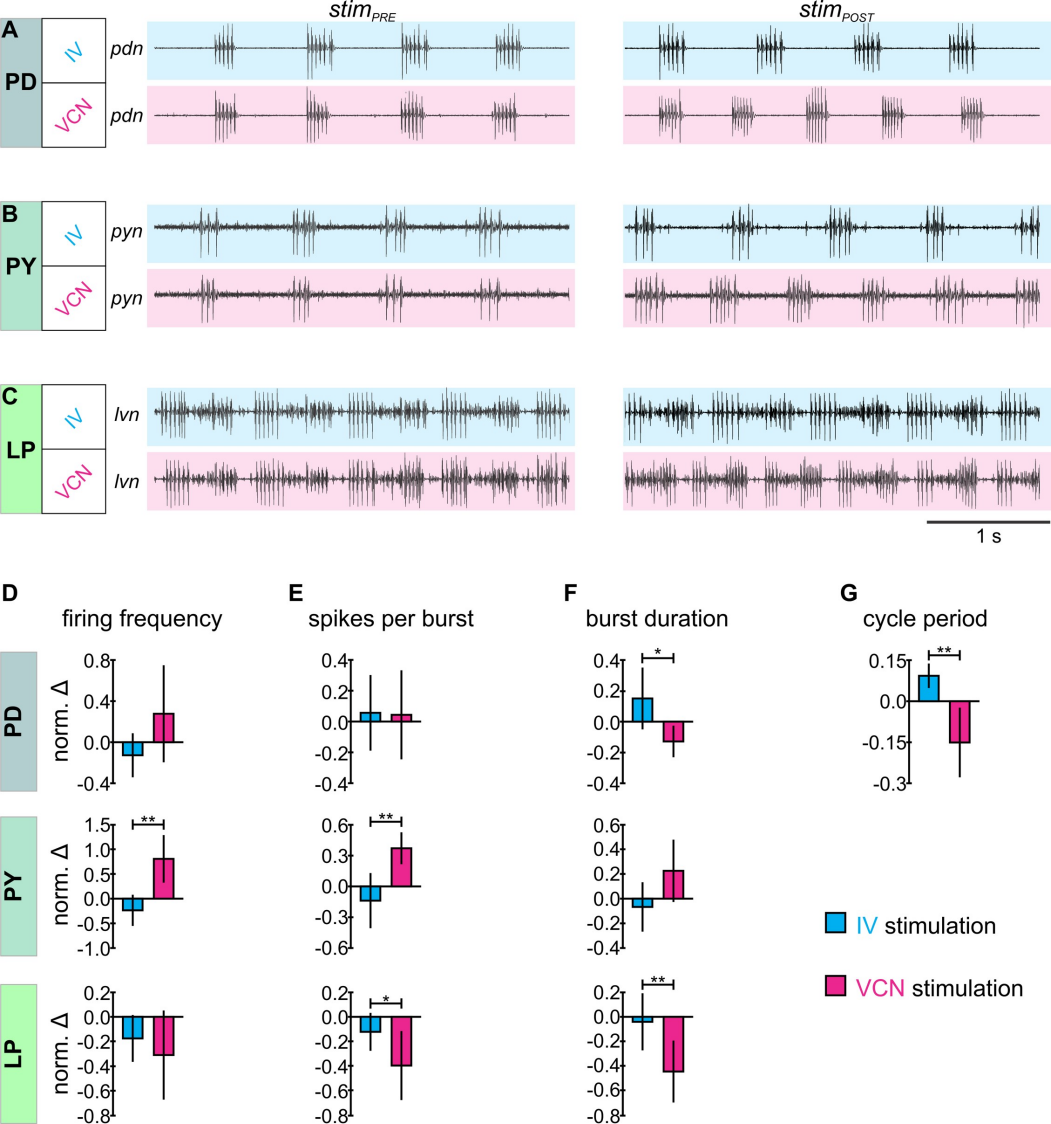
Unimodal chemosensory and mechanosensory inputs yield functionally different pyloric motor patterns. (A-C) Sample traces of extracellularly recorded PD, PY, and LP neuronal activity, respectively, immediately before (*stim*_*PRE*_, left) and after (*stim*_*POST*_, right) sensory stimulation for IV unimodal input, VCN unimodal input, and bimodal input, as indicated. The LP activity is the largest unit on *lvn*. (D-H) Quantification of pyloric rhythm activity in response to IV unimodal (cyan bars), VCN unimodal (magenta bars), and bimodal (dark gray bars) input for PD (top plots), PY (middle plots), and LP (bottom plots). Neuronal firing frequency (D), number of spikes per burst (E), and burst duration (F) were calculated separately for each neuron, while cycle period (G) is a measurement reflective of the whole pattern. Data (D-F) are mean ± SD of the normalized change in activity from *stim*_*PRE*_ to *stim*_*POST*_ (*N* = 8 for PD and LP, *N* = 7 for PY, *p < 0.05, **p < 0.01, ***p < 0.001, paired *t* test, the underlying data can be found in S1 Data). If not indicated, then no significance was found. IV, inferior ventricular neuron; LP, lateral pyloric; *lvn*, lateral ventricular nerve; PD, pyloric dilator; *pdn*, pyloric dilator nerve; PY, pyloric constrictor; *pyn*, pyloric constrictor nerve; VCN, ventral cardiac neuron.

Networks such as the one studied here are involved in the selection and maintenance of downstream motor activities, shaping the dynamics of ongoing motor programs [58,59]. They may be involved in decision-making when sensory conditions change [60,61]. Could the observed distinct combinations of CoG neurons be related to motor output? To test this, we monitored the activities of several identified STG motor neurons in both sensory conditions (Fig 8 and S1 Fig). We recorded the pyloric dilator (PD, Fig 8A) neurons, the pyloric constrictor (PY, Fig 8B) neurons, and the lateral pyloric (LP, Fig 8C) neuron. They together build the triphasic pyloric rhythm, with PD driving the rhythm as part of the pyloric pacemaker kernel and LP being the sole follower neuron that feeds back to the pacemakers [62–64]. The pyloric motor output is spontaneously active, making it amenable to detecting changes in upstream activity. Indeed, several motor pattern parameters were significantly different between IV and VCN stimulation, consistent with the hypothesis that changes in the CoG network have functional consequences for motor output. Most obviously, the cycle period of the pyloric rhythm was increased during IV stimulation, whereas it decreased during VCN stimulation (Fig 8G). This was likely caused by antagonistic effects on the PD pacemaker neurons, whose firing frequencies were reduced by IV stimulation and increased by VCN stimulation. The pyloric follower neurons PY and LP were also affected: PY had a significantly greater increase in its firing frequency (Fig 8D) and number of spikes per burst (Fig 8E) following VCN stimulation than following IV stimulation; LP had a significantly greater decrease in its number of spikes per burst (Fig 8E) and burst duration (Fig 8F) following VCN stimulation than following the IV stimulation.

In addition to their actions on the CoGs, both pathways have direct effects on the STG motor circuits via their axon projections to the STG. Direct effects are only described for the IVs (see [65]) and in this case include a rapid inhibition of the pyloric rhythm during the stimulation. We addressed this potential confounding issue with two approaches. One, since the direct effects seem to subside quickly, we excluded the first pyloric cycle after stimulation from the analysis (usually around 1–2 seconds). In addition, we ran an additional set of experiments in which the CoGs were removed, and only the direct effects of stimulating IV and VCN pathways (respectively) remained. Similar to before, we excluded 1–2 seconds immediately after the end of the stimulation from our analysis. Using the same measurements as were used in Fig 8 (firing frequency, number of spikes per burst, burst duration, and cycle period of the pyloric neurons), we found no differences when comparing before (*stim*_*PRE*_) and after (*stim*_*POST*_) stimulation (S2 Fig). Thus, the effects on the pyloric rhythm we observed with intact CoGs were likely mediated by the CoG neurons and not by direct sensory effects on the STG. It may remain unclear whether additional quickly dissipating sensory effects alter the extent of activation of the CoG neurons after the end of the stimulation. Nevertheless, this indicates that at least some of the effects on the CoG neurons are long lasting and persisted beyond the end of the sensory stimulation.

### Bimodal sensory input recruits a distinct combination of neurons

Sensory pathways were stimulated separately in the above experiments. During natural behaviors, however, sensory stimuli from multiple inputs may arrive concurrently. Although the IV and VCN modalities have never been examined in conjunction, they complement each other in vivo. The stomatogastric nervous system coordinates aspects of feeding in decapod crustaceans [66–68]. In this context, the IVs relay chemosensory information regarding potential food odor, and the VCNs activate with stomach distension as the crab ingests food; at this time, both IV and VCN pathways are active. To test whether concurrently arriving sensory stimuli are represented by a distinct network response, we carried out an additional set of experiments: we stimulated IV and VCN pathways individually (unimodal input) and simultaneously (bimodal input) and quantified activity maps across all three sensory conditions. Like the individual stimulations, bimodal stimulation yielded a spatially distributed network without clustering of excited and inhibited neurons (Fig 7A, right). Like the individual sensory conditions, simultaneous stimulation affected a substantial proportion of the CoG neurons. Across animals, 7.25% ± 3.89% did not respond to bimodal stimulation, whereas 59.19% ± 6.82% were excited and 33.56% ± 7.32% inhibited (*N* = 8, 5 crabs).

To test if different combinations of neurons were recruited in the bimodal situation, we tracked the identities and responses of all neurons individually and assessed how many neurons showed unique responses in one of the three conditions. For example, a neuron was classified as unique in the IV condition if it was inhibited by IV stimulation but excited by VCN and bimodal stimulation. Of the neurons, 25.14% ± 3.63% were unique to the IV condition, whereas 18.01% ± 3.9% were unique to the VCN condition and another 19.81% ± 5.14% to the bimodal condition (*N* = 6, 4 crabs).

**Fig 9:**
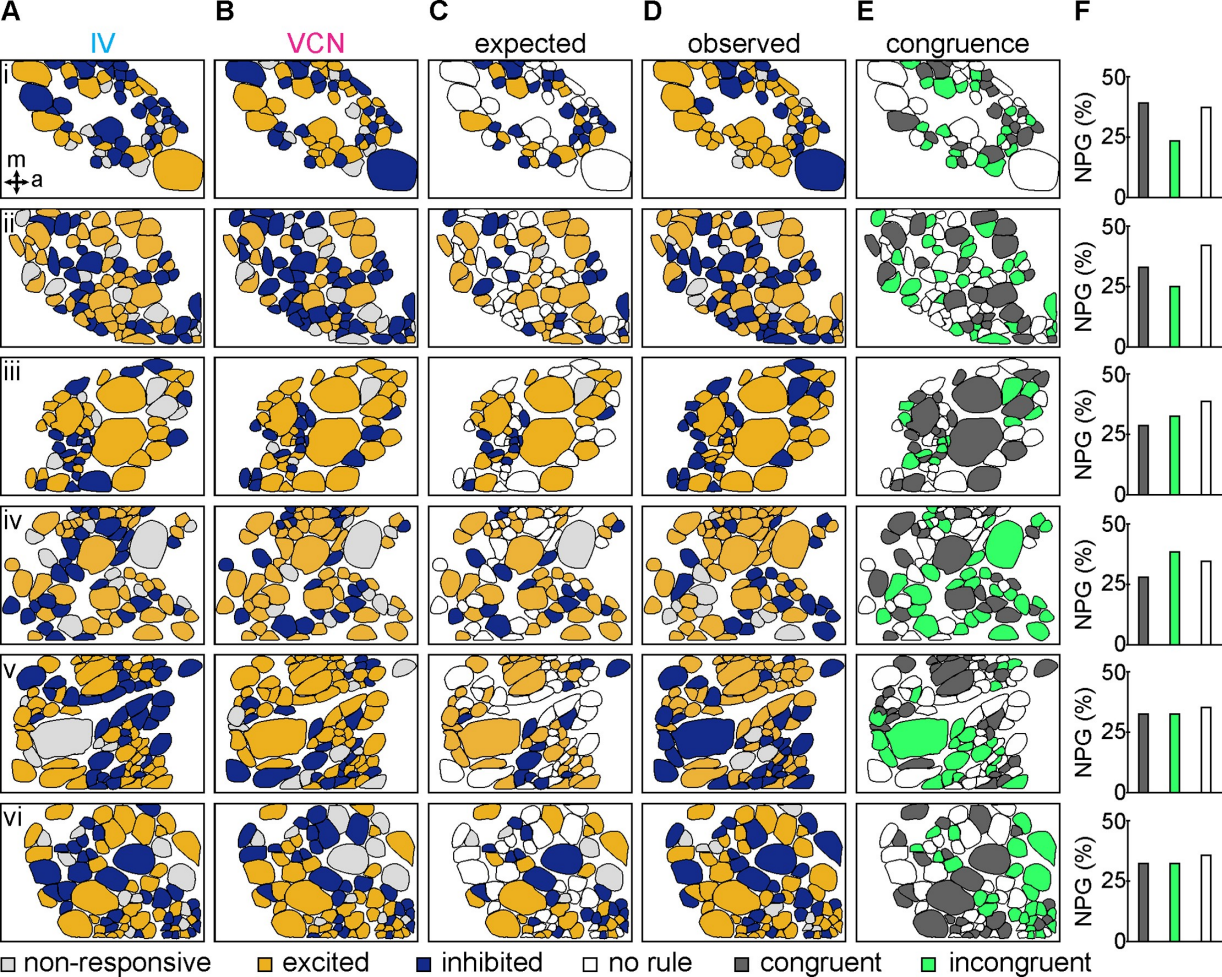
CoG network response to bimodal sensory stimulation differs from expected outcomes. (A and B) Maps of excited (yellow cell bodies), inhibited (blue cell bodies), and nonresponsive (light gray cell bodies) neurons across six ganglia (i–vi) during IV (A) and VCN (B) stimulation. (C) Map of expected outcomes for bimodal IV and VCN stimulation, based on additive responses ruled from the unisensory responses, and additional scenarios when no expectations could be formulated (“no rule,” white cell bodies). The set of expected outcomes for additive responses during bimodal stimulation of IV and VCN pathways was extrapolated based on additive responses as follows (see also Materials and methods): an excitatory response to both unimodal stimuli is expected to result in excitation during bimodal stimulation (IV_ex_ / VCN_ex_ = bimodal outcome [CO_ex_]). Similarly, an inhibitory response to each modality should yield an inhibited bimodal response (IV_inh_ / VCN_inh_ = CO_inh_). No response to either modality should result in no bimodal response (IV_NR_ / VCN_NR_ = CO_NR_), and an excitatory response to one unimodal stimulus and no response to the other would lead to an excitatory bimodal response (IV_ex_ / VCN_NR_ = CO_ex_; IV_NR_ / VCN_ex_ = CO_ex_). An inhibitory response to one unimodal input and no response to the other would result in an inhibitory bimodal response (IV_inh_ / VCN_NR_ = CO_inh_; IV_NR_ / VCN_inh_ = CO_inh_). Two scenarios (IV_ex_ / VCN_inh_ and IV_inh_ / VCN_ex_) cannot directly predict the additive outcome, because the potential outcome depends on the strengths of the excitation and inhibition. These scenarios are dubbed “no rule” (white). (D) Experimentally acquired neuronal responses during bimodal IV and VCN stimulation (observed outcomes). (E) Map of congruency between expected (C) and observed (D) outcomes, illustrating the agreement between the expected and observed cases. Dark gray: neurons whose observed and expected outcomes matched (“congruent”). Green: neurons whose expected and observed outcomes did not match (“incongruent”). White: neurons with no clear expected outcomes (“no rule”, IV_ex_ / VCN_inh_ and IV_inh_ / VCN_ex_), which was the case for 37.19% ± 2.87% of all neurons imaged (white cell bodies, *n* = 159; *N* = 6). (F) Proportion of neurons for each ganglion with congruent (dark gray) and incongruent (green) bimodal responses and when no rule applied and thus no expectations could be made (white), the underlying data can be found in S1 Data. IV, inferior ventricular neuron; NPG, number of neurons per ganglion; VCN, ventral cardiac neuron.

Bimodal sensory input can be perceived as a unique condition that is distinct from the experience of its unimodal inputs [5,69,70]. Little is known how such unique representations may be achieved in neural networks. The fact that about 19% of the CoG neurons were unique to the bimodal condition already indicated that part of the answer could lie in the combination of recruited neurons. However, if this was the case, one would also expect that the unique combination may not just be the sum of the representation of both inputs but instead be quite distinct from the individual responses. To test this, we generated a set of expectations for neuronal responses in the bimodal condition, based on the responses observed in each unimodal condition (see Materials and methods). We then compared the observed responses to the predicted responses. For about 37% of the neurons, no prediction could be made. This was the case if, for example, a neuron was excited by VCN but inhibited by IV stimulation. The bimodal response could thus not directly be predicted, as it would depend on the level of excitation and inhibition in each condition. For the about 63% of the cells for which we were able to create predictions, we found a mix of additive and nonadditive responses within the CoG network during the bimodal condition. The responses in the unimodal conditions were only predictive (congruent) for 32.19% ± 4.06% of neurons, whereas expectations were not predictive (incongruent) for 30.63% ± 5.44% of neurons (Fig 9). Consequently, for about half of the neurons in which expectations could be generated, the observed outcomes did not match the expected outcomes, indicating a significant reorganization of the CoG network activity when processing bimodal information. Thus, rather than a combined representation of the responses to individual IV and VCN stimuli, a new combination of responses emerged to represent the bimodal condition, providing further evidence for a combinatorial code that encodes sensory stimuli in CoG neurons.

## Discussion

We investigated how different sensory conditions are represented in a network participating in motor control [71,72]. We found processing to be spatially distributed within the network and involving the majority of the imaged neurons. Different sensory modalities were processed by a set of highly overlapping neurons, yet distinct combinations of neurons were recruited in each modality. This feature was consistent with almost no variability across animals, despite the rather large anatomical variability observed in this region [38]. We conclude that a combinatorial code is employed for sensory modality encoding in the CoG network—a coding scheme that may drive activity in the downstream pyloric motor circuit.

### Potential mechanisms for the encoding of multimodal sensory information

Several hypotheses exist for how neural networks encode multimodal information, following the concepts developed in unisensory areas. (1) Rate coding may encode sensory stimuli from different sense organs in the firing or burst frequencies of the involved neurons. Rate coding is mostly considered in unisensory parameter distinction like odorant intensity [73], tastant type [74], and many mechanosensory and proprioceptive sense organs that convey feedback magnitude in response to movements [75,76]. This is consistent with the idea that rate codes allow encoding of a large range of stimuli of the same type. In addition, rate coding has been suggested to be involved in cortical processing: visual-tactile information is differentiated via spike train dynamics in primary visual and somatosensory cortices [77], vestibular-visual information is involved in adaptive oculomotor responses [78], and visual-proprioceptive discriminations are made in the posterior parietal cortex [79]. Rate coding may also contribute to encoding of multimodal information in midbrain and brain stem: auditory and visual information yield disparate firing in the superior colliculus [80,81], and neurons in the solitary nucleus are suggested to use the specific timing of action potentials relative to one another (temporal coding) for taste and odorant discrimination [82]. A common argument for rate coding in these cases is that it may increase multimodal information capacity; i.e., rate coding allows encoding of a large set of parameters with fewer neurons while maintaining a broad and dynamic range of spike frequencies. (2) Alternatively, sensory information may be encoded in a largely overlapping population of neurons with distinct activation of the involved neurons (a combinatorial code). A combinatorial code may allow networks to make more robust distinctions when parameter space is limited [20], making it less suitable for encoding wide ranges of sensory activities but a prime candidate for distinguishing between categorically different stimuli, such as sensory modalities. Most evidence for combinatorial coding schemes comes from studies of unisensory stimuli, though, such as odor discrimination and object localization [73,83,84], or from studies of multisensory encoding at the single-cell or coarse, large-scale network levels. In the basal ganglia, for example, distinct striatal subpopulations were described that responded either only to tactile stimuli or to both tactile and visual inputs [6], suggesting the presence of a combinatorial code. Few studies have addressed how multisensory integration is achieved at the network level and how these network responses may be involved in controlling behavioral output.

### A combinatorial code of CoG neurons differentiates between sensory modalities

Premotor control networks have long been known to process sensory information from different sense organs [8,13,85]. In vertebrates, brain stem studies in lamprey, such as visual- and electrosensory-guided gaze reorientation [11] and reticulospinal gating of steering [36], as well as similar findings in cat superior colliculus [85], exemplify this feature. In leech, contributions of distinct but overlapping subsets of neurons are involved in the decision whether to swim or crawl [60,61], whereas in *Aplysia* and *Tritonia*, network dynamics change during the execution of a locomotor program and during behavioral sensitization [58,59]. Our approach allowed us to monitor network activity and motor responses separately, because motor and premotor neurons reside in spatially distinct regions. In the stomatogastric nervous system, sensory information from multiple sensory modalities and modulatory pathways activates a small set of projection neurons residing within the CoGs. These projection neurons then innervate the central pattern generators in the downstream STG, where they initiate and modulate distinct variants of gastric mill and pyloric motor patterns [40,41,86–88]. Previous studies have proposed that the combination of activated descending projection neurons differs between modalities and plays a role in motor pattern selection [41]. Conversely, changing the burst structure and firing patterns of the same neurons also elicits different variants of the gastric mill motor pattern [71,86,89]. Our findings indicate that the projection neurons are part of a larger CoG network that processes sensory information from multiple modalities. While the true connectivity between neurons in this network and the projection neurons cannot be assessed due to the sheer number of involved neurons, our CoG connectivity density and global efficiency measurement suggest a coherent network for processing of the two modalities. We find that the most parsimonious interpretation of our data is that the difference between the two modalities is encoded in the combination of excited and inhibited neurons. Per definition, such a combinatorial code takes into account which neurons participate in, and contribute to, the differentiation between sensory stimuli but also the activity patterns of the involved neurons. Inhibited neurons are essentially prevented from participating in the network response or strongly reduced in their contribution, whereas excited neurons contribute to the population response. With only a few hundred neurons [38], the CoG network thus employs a combinatorial scheme to dynamically change CoG neuron responses in different sensory conditions.

While not tested here, there are several other modalities that are processed by the CoG neurons, including proprioceptors like muscle stretch receptors. We are not aware of any other mechanosensory and chemosensory pathways that converge onto the CoGs that could be used to test intramodality differences. However, within each modality, a wide range of stimulus properties such as strength (frequency) and stimulus patterns elicit very similar motor patterns [42,43]. Across modalities, the motor patterns are distinct. While this does not necessarily mean that CoG neuron responses remain constant within all modalities, it does indicate that modalities elicit categorical responses rather than continuous ones.

The CoG is a hub with neurons that project to the STG, the brain, and thoracic ganglion [38,90]. The STG is thus not the only neural structure postsynaptic to CoG projection neurons. Given the large number of multimodal neurons that respond to both IV and VCN stimulation and the coherent processing within the CoG network, it seems reasonable to assume that the projection neurons represent the output layer of this network. If this were the case, then the CoG network would act to preprocess and distinguish the sensory modalities before this information was sent to other areas in the nervous system. For the STG, the identified descending modulatory projection neurons would then elicit the differences we observed in the pyloric motor pattern.

### Bimodal stimuli are represented by a new combination of recruited neurons

Theoretical and perceptual learning studies suggest that bimodal conditions yield new percepts that are not simply the sum of their unimodal components [5,91,92]. Part of a larger binding problem, it is thought that if distinct sensory modalities are active within a particular temporal window, they may be perceptually bound as a single event. In terms of motor control, bimodal input can lead to enhanced or new behavioral responses that are distinct from the responses to the individual sensory modalities. Using visual and mechanosensory cues for guidance, fly odor tracking, for example, is significantly enhanced during flight, and bimodal processing is a prerequisite for this particular behavior [93]. Similarly, localization of spatiotemporally concordant stimuli is achieved via combined auditory and visual input [94].

Little is known about how different percepts and behavioral responses are generated and what network responses underlie these processes. Most studies of multisensory processing, in particular those done in the context of motor control, consider one modality at a time. To encode a unique condition, network responses have to be distinct from the unimodal responses. Super- and subadditive responses to bimodal input are quite common features in individual neurons, for example, in integrating and nonintegrating interneurons of the superior colliculus [81]. This contrasts with some studies, such as moth tracking behavior [95], in which multimodal responses are the linear sum of two unimodal inputs. In the latter case, however, the bimodal condition serves the same behavioral function as the unimodal conditions—to strengthen the robustness of the behavior through redundancy in sensory input. We found that the CoG network response to the bimodal condition was distinct not only from the individual unimodal network responses but also from the expected summation of both. Specifically, more than half of the network activity was incongruent with the expected bimodal responses, demonstrating that the bimodal condition is not an additive function of unimodal input. More importantly, the identities of CoG neurons recruited in the two individual sensory conditions were different from those in the bimodal condition, consistent with a new combination of neurons that encodes this condition. Functionally, the bimodal condition is indeed different from the individual conditions in the context of the behavior driven by the stomatogastric system: when an animal encounters food, this will first activate the chemosensory IV pathway, and IV activity will likely continue as long as food is ingested. Once food enters the stomach, the mechanosensory VCN pathway will activate in addition to the chemosensory pathway. This bimodal condition thus indicates both that food is ingested and that food is filling the stomach at the same time. However, stomach distention can outlast the availability of food, for example, when a predator interrupts the meal or when all food is ingested. This will lead to a loss of chemosensory activation but maintains mechanosensory feedback. This sequence from unimodal chemosensory to bimodal and unimodal mechanosensory may allow the animal to differentiate an initial unfed and feeding state from a later fed but still food-processing state. For the pyloric filter apparatus, transitioning between these states may be accompanied with distinct filter movements. Finally, transitioning to the nonfeeding state with a full stomach later on may also be important for other behaviors, such as locomotion and decisions about flight or fight, which are mediated by the concerted actions of the brain and thoracic ganglia, both of which receive innervation from the CoG network [38,90].

### Implications for multimodal encoding and motor pattern selection in higher-order brain areas

We provide evidence that distinct sensory modalities are encoded by a combinatorial code in a sensorimotor system with direct link between sensory input and motor output. Our results, however, may also provide a mechanism by which more complex networks could encode bimodal sensory information or, more generally, converging neuronal information. This is reinforced by the more recent evidence for combinatorial coding in higher-order brain regions, like the superior colliculus [11,85], basal ganglia [6], and even cortical areas [20]. The network studied here is dedicated to processing of sensory information from several modalities and involved in controlling behavioral output [41]. Motor circuits such as central pattern generators and their control circuits are quite distinct in function from neocortical circuits. Nevertheless, they also share several profound similarities, including spontaneous activity waves, rich dynamics and plasticity, powerful modulation, and engagement by sensory pathways [96]. Given that cortical circuits also share several anatomical and biophysical properties with downstream networks, such as repetition of small circuit modules, it is conceivable that the mechanisms present to distinguish and encode different sensory modalities foretell the basic principles of multimodal processing in cortical circuits or even perception.

### Conclusions

How neuronal networks process multiple sensory inputs simultaneously remains an enigmatic problem. In a sensorimotor system, different modalities are processed by a largely overlapping set of neurons, and the differences between modalities are encoded in the combination of recruited neurons.

## Materials and methods

### Animals

Adult Jonah crabs (*Cancer borealis*) were acquired from The Fresh Lobster Company (Boston, MA, United States) or Ocean Resources (Sedgwick, ME, US). Crabs were kept in tanks with artificial seawater (salt content approximately 1.025 g/cm^3^; Instant Ocean Sea Salt Mix, Blacksburg, VA, US) at a temperature of 11°C and a 12-hour light–dark cycle. Animals were anesthetized on ice for 30 minutes before dissection. We used isolated nervous systems to perform all of our experiments [97].

### Solutions and reagents

For electrophysiological experimentation, nervous systems were continuously superfused (7–12 ml/min) with chilled (10–13°C) *C*. *borealis* saline consisting of (all from Sigma, St. Louis, MO, US) NaCl, 440 mM; KCl, 11 mM; MgCl_2_*6H_2_0, 26 mM; CaCl_2_, 13 mM; trisma base, 10 mM; maleic acid, 5 mM (pH 7.4–7.6).

### Application of voltage-sensitive dye

The lipophilic voltage-sensitive dye Di-4-ANEPPDHQ (Thermo Fisher Scientific) was bath-applied to stain neuronal membranes [47]. Stock solutions (5 mM) in dimethyl sulfoxide were aliquoted for single use and kept at −20°C. Immediately before application, solutions were diluted 1:1 with pluronic acid F-127 (20% solution; Biotium, Hayward, CA, US) dimethyl sulfoxide solution and mixed with saline to a final concentration of 50 μM. A petroleum jelly well was built around the desheathed CoG (S1 Fig), and the dye was applied for 30–60 minutes, after which the petroleum jelly well was removed, and the preparation was continuously superfused with cooled (10–13°C) saline for the remainder of the experiment.

### Stimulation of sensory pathways

Mechanosensory: VCNs were activated by stimulating the *dpon* or ventral cardiac nerve (*vcn*) extracellularly with single stimulus trains of 1 Hz or 15 Hz stimulation frequency, following parameters established previously and mimicking mechanical stimulation of the VCN [42]. Chemosensory: The IVs were activated by stimulating the *ivn* extracellularly with single stimulus trains of 1 Hz or 40 Hz stimulation frequency, at the upper end of the physiological IV activity range [43]. Petroleum jelly wells were built around the *dpon* and the *ivn*. For each nerve, one of two stainless steel stimulation electrodes was placed inside the petroleum jelly compartment, and the other was placed outside. All stimuli (regardless of stimulation frequency) were presented continuously for 6 seconds with 1 ms pulse durations. Stimulus amplitude (voltage) was determined separately in each experiment by determining the minimum threshold for a response in the pyloric motor neurons. For experimentation, twice the threshold voltage was used. Stimulation commands were generated in Spike2 (version 7.13; Cambridge Electronic Design, Cambridge, United Kingdom) and converted to analog signals via a Power 1401 digital-analog converter (Cambridge Electronic Design, Cambridge, UK).

### Optical imaging

For recording fluorescence changes, the MiCam02 imaging system and BV-ANA software (Brain-Vision Analyzer, Version 11.08.20; SciMedia, Tokyo, Japan) were used (spatial resolution = 192 × 128 pixels; frame rate = 250 frames/second). Individual recordings lasted 20 seconds and were repeated many times in a given experiment. Excitation light was provided by a narrowband LED with 525 nm (CoolLED, Yorktown Heights, NY, US), and fluorescence emission was filtered through a quadband filter cube (Semrock, Rochester, NY, US). Excitation light intensities varied and were adjusted to the individual preparation. We used a 20× objective (UMPlanFL N, NA 0.30, WD 3.3 mm, cc = water; Olympus, Tokyo, Japan) mounted on an upright epifluorescence microscope (modified BX51, Scientifica, East Sussex, UK).

### Extracellular and intracellular electrophysiology

Spike activities of the pyloric neurons were acquired via extracellular recordings of motor nerves posterior to the STG (S1 Fig, schematic): we used the lateral ventricular nerve (*lvn*) for LP activity, the pyloric dilator nerve (*pdn*) for PD activity, and the pyloric constrictor nerve (*pyn*) for PY activity. Note that the *pdn* and *pyn* contain multiple PD and PY neuron units, respectively, and we did not separate out these neurons individually. We used petroleum jelly wells and subsequent measurements of field potential changes between two stainless steel wires (one inside and one outside of each well) to extracellularly record action potentials. The differential signal was recorded, filtered, and amplified with an AC differential amplifier (A-M Systems Modell 1700, Carlsborg, WA, US). Files were recorded, saved, and analyzed using Spike2 software.

Standard intracellular recording techniques were used for comparison of intracellular and optical signals recorded from CoG neurons [98]. CoG somata were visualized with white light transmitted through a dark field condenser (Nikon, Tokyo, Japan) to facilitate placement of the recording electrode. Intracellular recordings were obtained using 20–30 MΩ glass microelectrodes (Sutter 1000 electrode puller, Novato, CA, US) filled with a 0.6 M K_2_SO_4_ + 20 mM KCl solution. Signals were recorded in current-clamp configuration and amplified with a BA-1S Intracellular Bridge Mode Amplifier (NPI Electronic GmbH, Tamm, Germany). Files were recorded and analyzed at a sampling rate of 10 kHz with Spike2 software.

### Data analysis and figure construction

Imaging and electrophysiological data were analyzed with the Brain-Vision Analyzer software (BVAna SciMedia, Tokyo, Japan), Spike2 (version 7.13; CED), and custom-made MATLAB (version R2014b, MathWorks) scripts. Final figures were prepared with MATLAB and CorelDraw (version X7 for Windows, http://www.coreldraw.com). For spreadsheet analysis, Excel (version 2010–2013 for Windows, Microsoft) and R (version 3.3.1, The R Foundation for Statistical Computing) were used. Data are given as mean ± SD unless otherwise noted. “N” denotes the number of ganglia used; “n” denotes the number of neurons or trials. For some animals, more than one ganglion was used (each stomatogastric nervous system has two CoGs). Unless otherwise stated, with *N* = 12, 9 animals were used; with *N* = 11, 9 animals were used; with *N* = 6, 4 animals were used; with *N* = 8, 5 animals were used; and with *N* = 19, 14 animals were used. Significance is indicated using *(*p* < 0.05), **(*p* < 0.01), and ***(*p* < 0.001). Statistical tests were paired *t* test, Pearson correlation coefficient, two-sample K-S test, and Tukey-Kramer test in conjunction with one-way ANOVA. Details of each individual test used can be found in the figure legends to which they pertain.

### Imaging analysis

For comparing changes in fluorescence of the entire imaged region (spatial averaging), we used the average whole-field change in fluorescence during 6-second stimulation and subtracted with the 6-second prestimulation (Δ fluorescence, Fig 1C). This included the averaged signals of all imaged neurons. CoG neurons are found in multiple focal planes, but as one moves from dorsal to ventral, the somata distribution tends to spread outward. This alleviates potential detection of optical signals from neurons above or below the imaged focal plane. In addition, CoG somata are large, which further reduces bleed-through from other cells. We had previously shown that optical signals are strongest when a neuron is in focus.

We measured firing frequencies of all imaged neurons to detect sensory-induced responses in CoG neurons. Voltage-sensitive dyes are sufficiently fast to detect sub-millisecond events [99]. Individual cell traces and contours were extracted using BV-ANA software, and further analysis was executed in MATLAB. Traces were processed with a drift removal based on six-order polynomial fit, and spike threshold was based on the median of the signal [100]. In addition, high-pass filtering was used to separate fast action potentials from slower and often larger changes in membrane potential. Since spike amplitude varied across neurons, high-pass filters were adjusted separately for each neuron by assessing the maximum difference between slow and fast changes in membrane potential.

For rate-coding analyses (Fig 5), we calculated in each experiment for each condition the ratio between spike frequency during 6 second stimulation (*stim*_*ON*_) and 6 seconds immediately preceding stimulation (*stim*_*PRE*_). For each condition, the distribution of the frequency ratios of all neurons in a given preparation was plotted as a histogram (bin size: 0.1). Distributions were normalized to the maximum count for each experiment. Frequency ratio distributions were then averaged across preparations and tested for differences between conditions using the two-sample K-S test. Ratios > 1 indicate neuronal activity increases (excitation), ratios < 1 indicate activity decreases (inhibition), and ratios = 1 indicate no change in spike frequency. To avoid division by zero, if a neuron was not spontaneously active, it was omitted from the analysis, although this was rare. In addition, we calculated the distribution of the frequency difference during stimulation (*stim*_*ON*_) and immediately preceding stimulation (*stim*_*PRE*_) and tested for differences between conditions using K-S test.

To categorize individual neurons as “excited,” “inhibited,” or “nonresponsive,” we used the difference between the number of spikes during stimulation and prestimulation (Fig 6C–6G). Pairwise comparisons for these categorizations were made between IV and VCN conditions for each CoG and subsequently averaged across ganglia.

Neurons were defined as unimodal when their activities changed in response to stimulation of only one of the two sensory modalities. Neurons that responded to both sensory modalities were defined as multimodal. Neurons that did not respond to either stimulation were defined as “nonparticipant” (Fig 6A).

For the spatial distribution analysis (Fig 7), we calculated the angle from the Cartesian coordinates and used the angular variance as a measure of directional variability. To normalize the distances for each ganglion, we determined a polygon contour around the outer neurons, found the centroid, and calculated the ratio between the distance from neuron to centroid and distance from border to centroid, d_ratio_ = d(neuron,centroid) / d(border, centroid).

To identify the proportion of neurons that had a unique response to one of the sensory conditions, we calculated the proportion of CoG neurons that share a similar response type to two of the sensory conditions, whereas the response to the remaining condition differs (unique). For example, a neuron that was excited by IV stimulation but inhibited by both VCN and bimodal stimulations would be a unique response for the IV condition, whereas a neuron that was excited by IV, VCN, and bimodal stimulations is not a unique participant and was omitted from the analysis.

For the expected map responses (congruency under bimodal conditions, Fig 9), we considered nine response combination scenarios, given the three potential neuronal responses (excited, inhibited, and nonresponsive) to unimodal sensory input. Seven of these can be used to generate clear expectations of bimodal stimulation effects and to test whether neuronal responses to bimodal stimulation are the additive result of the responses observed during unimodal stimulation: excitatory responses to both unimodal inputs will summate to an excitatory bimodal response (IV_ex_ / VCN_ex_ = CO_ex,_). An inhibitory response to both unimodal inputs will yield an inhibited bimodal response (IV_inh_ / VCN_inh_ = CO_inh_), and no response to either stimulus should not result in a response to bimodal stimulation (IV_NR_ / VCN_NR_ = CO_NR_). An excitatory response to one unimodal input and no response to the other will summate to an excitatory response for bimodal stimulation (IV_ex_ / VCN_NR_ = CO_ex_; IV_NR_ / VCN_ex_ = CO_ex_), and an inhibitory response to one input with no response to the other will yield an inhibitory bimodal response (IV_inh_ / VCN_NR_ = CO_inh_; IV_NR_ / VCN_inh_ = CO_inh_). Two scenarios (IV_ex_ / VCN_inh_ and IV_inh_ / VCN_ex_) are inconclusive, because the additive outcome cannot directly be predicted as it depends on the weighting of excitation and inhibition. We used spatial maps of the previously characterized neuronal responses to create spatial maps of the expected outcomes (Fig 9). A Boolean-based comparison of the additive expectations with those data acquired for multimodal responses was used to demonstrate the proportion neurons that did not respond additively (“no match”). This analysis produces a conservative estimate of which neurons show dramatically distinct responses in the bimodal condition. It was chosen over creating expectations maps using firing frequency changes, which is more sensitive to small changes but may also produce false positives, since neuronal firing frequency responses (F/I curves) are rarely linear.

### Motor pattern analysis

For the motor neuron analysis, a custom-written program for Spike2 was used to determine pyloric rhythm activities. Pyloric neuron activity measurements were calculated as follows: firing frequency as the number of action potential spikes divided by the interval of time (seconds) measured; burst duration as the time (seconds) from the first to the last spike in a burst; spikes per burst as the number of spikes counted in a burst; and the cycle period was determined by calculating period between the onset of two successive PD neuron bursts. We did not separate multiple PD neurons (2 found on the *pdn*) or PY neurons (4 found on *pyn*) when calculating these parameters. Measurements were plotted as the normalized change (norm. Δ) between *stim*_*PRE*_ and *stim*_*POST*_ (norm. Δ = [*stim*_*POST*_ − *stim*_*PRE*_] / *stim*_*PRE*_, Fig 8). All procedures were the same for experiments testing the direct effects of sensory stimuli on pyloric motor output, except that in these experiments, the CoG influences were removed by transecting the inferior esophageal nerve (*ion*) as well as the superior esophageal nerve (*son*) between the *dpon* and the CoGs (see S1 Fig for nerves). This procedure bisects the IV and VCN axons that project to the CoGs, and it removes all CoG influences on the STG. In contrast, the direct axon connections between IVs and STG, as well as between VCN and STG, are maintained. As an alternative, high-potassium saline (120 mM KCl) was applied to the CoG to block action potentials in the CoGs.

### Functional connectivity analysis

For establishing the functional connectivity, we first determined a coherence matrix between the fluorescence traces (time series, *V*) of all neurons in a given ganglion using Pearson’s correlation. Each element of the coherence matrix (K*x*K) is calculated using

$$\rho_{ij}=\frac{\sum_{t}\left(V_{i}(t)-\bar{V_{i}})(V_{j}(t)-\bar{V_{j}}\right)}{\sqrt{\sum_{t}{\left(V_{i}(t)-\bar{V_{i}}\right)}^{2}{\left(V_{j}(t)-\bar{V_{j}}\right)}^{2}}},$$

where *ρ*_*ij*_ is the correlation coefficient between neurons *V*_*i*_ and *V*_*j*_, $\bar{V_{i}}$ and $\bar{V_{j}}$ are the means, K is the number of neurons in a given preparation, and *t* is the time. Each coherence matrix was thresholded to create an adjacency matrix. Two neurons *i* and *j* are considered functionally connected if *ρ*_*ij*_ is greater than a threshold. We opted for a fixed threshold to determine the adjacency matrix, which was empirically set to 0.45 and *p* < 0.05 [49]. Adjacency matrix is a symmetric matrix, and elements can be either 1 or 0. A value of 1 indicates that there is a functional connection (link) between neurons (node), and a value of 0 indicates no connection (link). We considered two network metrics:

- Connectivity density defined as the proportion of connections (links or edges, *m*) that exists relative to the number of possible connections of an undirected network [101,102].
  $$Density=\frac{2m}{K(K-1)}$$
  This measure gives an indication of how well connected a network is. A connectivity density of 1 means that all potential connections exist; i.e., each neuron is connected to all other neurons.
- Global efficiency, *E*_*Global*_ defined as the inverse of the harmonic mean of the shortest path length *L*_*ij*_ (smallest number of edges that connect two nodes) between each pair of nodes [52,53].
  $$E_{Global}=\frac{1}{K(K-1)}\sum_{i\neq j\in Graph}\frac{1}{L_{ij}}$$

It is a measure of how efficiently information is exchanged over the network, resulting in a measure of the global efficiency of parallel information transfer in the network.

## Supporting information

S1 Fig VSD imaging in a multifunctional motor system. (A) The stomatogastric nervous system consists of four ganglia: the STG, the OG, and the bilaterally paired CoGs (only right CoG shown here). The chemosensory IVs (cyan) descend from the SoG (“brain”) and innervate the CoGs via the *ivn*, *ion*, and *son* and the STG via the *stn*. The mechanosensory VCNs (magenta) innervate the CoGs via the *dpon*. VSD imaging was used to monitor CoG neuronal activity. (B) While CoG neurons project to many different locations throughout the nervous system such as the TG and the brain (SoG), a subset of the CoG neurons localized in the medial-posterior area of the ganglion (dotted box) project to downstream motor circuits (arrow) in the STG. (C) Example image of neuronal cell bodies in the CoG area that contains descending projection neurons that control the STG motor circuits. About 80 distinct cell bodies are distinguishable via their brightly fluorescing cell boundaries. All neurons in a single focal plane were imaged simultaneously. (D) Optical trace from a single neuron in (C), showing the change in spike activity in response to VCN stimulation. (E) Sample traces of the downstream pyloric motor neurons recorded extracellularly from motor nerves posterior to the STG. Action potential (spike) information was attained for the LP neuron, the PY neurons, and the PD neurons via recordings of the *lvn*, the *pyn*, and the *pdn*, respectively. CoG, commissural ganglion; *dpon*, dorsal posterior esophageal nerve; *ion*, inferior esophageal nerve; IV, inferior ventricular neuron; *ivn*, inferior ventricular nerve; LP, lateral pyloric; *lvn*, lateral ventricular nerve; PD, pyloric dilator; *pdn*, pyloric dilator nerve; PY, pyloric constrictor; *pyn*, pyloric constrictor nerve; OG, esophageal ganglion; SoG, supraesophageal ganglion; *son*, superior esophageal nerve; STG, stomatogastric ganglion; *stn*, stomatogastric nerve; TG, thoracic ganglion; VCN, ventricular cardiac neuron; VSD, voltage-sensitive dye.
(TIF)

S2 Fig Direct influences of sensory input onto STG do not yield lasting effects on the pyloric rhythm. (A-D) Quantification of pyloric rhythm activity before (dashed bars, *stim*_*PRE*_) and after (solid bars, *stim*_*POST*_) IV stimulation (cyan bars) and VCN stimulation (magenta bars) for PD (top plots), PY (middle plots), and LP (bottom plots). Neuronal firing frequency (A), number of spikes per burst (B), and burst duration (C) were calculated separately for each neuron, while cycle period (D) is a measurement reflective of the whole rhythm. Data are mean ± SD. Comparisons were made within modalities, and no differences were found between *stim*_*PRE*_ and *stim*_*POST*_ activity (paired *t* test; no significance; *N* = 8 ganglia, 5 crabs, for PD; *N* = 7 ganglia, 4 crabs, for PY; and *N* = 7 ganglia, 6 crabs, for LP). CoG, commissural ganglion; IV, inferior ventricular neuron; LP, lateral pyloric; PD, pyloric dilator; PY, pyloric constrictor; STG, stomatogastric ganglion; VCN, ventricular cardiac neuron.
(TIF)

S1 Data (XLSX)

## Abbreviations

CoG: commissural ganglion
IV: inferior ventricular neuron
K-S: Kolmogorov-Smirnov
LP: lateral pyloric
n.s.: not significant
PD: pyloric dilator
PY: pyloric constrictor
STG: stomatogastric ganglion
VCN: ventral cardiac neuron

## References

[1] Ghazanfar AA, Schroeder CE. Is neocortex essentially multisensory? Trends Cogn Sci. 2006;10: 278–285. 10.1016/j.tics.2006.04.008 16713325

[2] Schroeder CE, Foxe J. Multisensory contributions to low-level, “unisensory” processing. Curr Opin Neurobiol. 2005;15: 454–458. 10.1016/j.conb.2005.06.008 16019202

[3] Rauschecker JP, Tian B, Hauser M. Processing of complex sounds in nonprimary auditory cortex of the rhesus monkey. Sci (New York, NY). 1995;268: 111–114. 10.1126/science.7701330 7701330

[4] Ehmer B, Gronenberg W. Segregation of visual input to the mushroom bodies in the honeybee (Apis mellifera). J Comp Neurol. Wiley Subscription Services, A Wiley Company; 2002;451: 362–373. 10.1002/cne.10355 12210130

[5] Driver J, Noesselt T. Multisensory interplay reveals crossmodal influences on “sensory-specific” brain regions, neural responses, and judgments. Neuron. Elsevier; 2008;57: 11–23. 10.1016/j.neuron.2007.12.013 18184561 PMC2427054

[6] Reig R, Silberberg G. Multisensory Integration in the Mouse Striatum. Neuron. The Authors; 2014;83: 1200–1212. 10.1016/j.neuron.2014.07.033 25155959 PMC4157575

[7] Getting PA, Dekin MS. Mechanisms of pattern generation underlying swimming in Tritonia. IV. Gating of central pattern generator. J Neurophysiol. 1985;53: 466–480. Available from: http://www.ncbi.nlm.nih.gov/entrez/query.fcgi?cmd=Retrieve&db=PubMed&dopt=Citation&list_uids=2984350 10.1152/jn.1985.53.2.466 2984350

[8] Meredith MA, Stein BE. Descending efferents from the superior colliculus relay integrated multisensory information. Science. 1985;227: 657–9. 10.1126/science.3969558 3969558

[9] Kozlov A, Huss M, Lansner A, Kotaleski JH, Grillner S. Simple cellular and network control principles govern complex patterns of motor behavior. Proc Natl Acad Sci. 2009;106: 20027–20032. 10.1073/pnas.0906722106 19901329 PMC2785286

[10] Juvin L, Grätsch S, Trillaud-Doppia E, Gariépy J-F, Büschges A, Dubuc R. A Specific Population of Reticulospinal Neurons Controls the Termination of Locomotion. Cell Rep. 2016;15: 2377–2386. 10.1016/j.celrep.2016.05.029 27264174

[11] Kardamakis AA, Pérez-Fernández J, Grillner S. Spatiotemporal interplay between multisensory excitation and recruited inhibition in the lamprey optic tectum. Elife. 2016;5 10.7554/eLife.16472 27635636 PMC5026466

[12] Stein W, Schmitz J. Multimodal convergence of presynaptic afferent inhibition in insect proprioceptors. J Neurophysiol. 1999;82: 512–514. Available from: http://www.ncbi.nlm.nih.gov/entrez/query.fcgi?cmd=Retrieve&db=PubMed&dopt=Citation&list_uids=10400981 10.1152/jn.1999.82.1.512 10400981

[13] Stein W, Buschges A, Bassler U. Intersegmental transfer of sensory signals in the stick insect leg muscle control system. J Neurobiol. 2006;66: 1253–1269. Available from: http://www.ncbi.nlm.nih.gov/entrez/query.fcgi?cmd=Retrieve&db=PubMed&dopt=Citation&list_uids=16902990 10.1002/neu.20285 16902990

[14] Burrows M, Pflüger H-J. Positive feedback loops from proprioceptors involved in leg movements of the locust. J Comp Physiol A Sens Neural Behav Physiol. 1988;163: 425–440.

[15] Stein BE, Arigbede MO. Unimodal and multimodal response properties of neurons in the cat’s superior colliculus. Exp Neurol. Academic Press; 1972;36: 179–196. 10.1016/0014-4886(72)90145-8 4558413

[16] Stein BE, Stanford TR. Multisensory integration: current issues from the perspective of the single neuron. Nat Rev Neurosci. 2008;9: 255–266. 10.1038/nrn2331 18354398

[17] Meredith MA, Stein BE. Visual, auditory, and somatosensory convergence on cells in superior colliculus results in multisensory integration. J Neurophysiol. 1986;56: 640–662. doi:citeulike-article-id:844215 10.1152/jn.1986.56.3.640 3537225

[18] Meredith MA, Nemitz JW, Stein BE. Determinants of multisensory integration in superior colliculus neurons. I. Temporal factors. J Neurosci. 1987;7: 3215–29. doi:citeulike-article-id:409430 3668625 10.1523/JNEUROSCI.07-10-03215.1987 PMC6569162

[19] Pennartz CMA. Identification and integration of sensory modalities: Neural basis and relation to consciousness. Conscious Cogn. 2009;18: 718–739. 10.1016/j.concog.2009.03.003 19409812

[20] Osborne LC, Palmer SE, Lisberger SG, Bialek W. The Neural Basis for Combinatorial Coding in a Cortical Population Response. J Neurosci. 2008/12/17 ed. 2008;28: 13522–13531. 10.1523/JNEUROSCI.4390-08.2008 19074026 PMC2693376

[21] Rohe T, Noppeney U. Distinct Computational Principles Govern Multisensory Integration in Primary Sensory and Association Cortices. Curr Biol. Elsevier Ltd; 2016;26: 509–514. 10.1016/j.cub.2015.12.056 26853368

[22] Xin Y, Weiss KR, Kupfermann I. An identified interneuron contributes to aspects of six different behaviors in Aplysia. J Neurosci. 1996;16: 5266–79. 8756454 10.1523/JNEUROSCI.16-16-05266.1996 PMC6579297

[23] Berkowitz A, Roberts A, Soffe SR. Roles for multifunctional and specialized spinal interneurons during motor pattern generation in tadpoles, zebrafish larvae, and turtles. Front Behav Neurosci. 2010/07/16 ed. 2010;4: 36 10.3389/fnbeh.2010.00036 20631847 PMC2903196

[24] Marder E, Thirumalai V. Cellular, synaptic and network effects of neuromodulation. Neural Networks. 2002;15: 479–493. Available from: http://www.ncbi.nlm.nih.gov/entrez/query.fcgi?cmd=Retrieve&db=PubMed&dopt=Citation&list_uids=12371506 12371506 10.1016/s0893-6080(02)00043-6

[25] Harris-Warrick RM, Johnson BR. Checks and balances in neuromodulation. Front Behav Neurosci. Frontiers Media SA; 2010;4: 1–9. 10.3389/neuro.08.001.2010 20700503 PMC2917248

[26] Nusbaum MP, Beenhakker MP. A small-systems approach to motor pattern generation. Nature. 2002;417: 343–350. Available from: http://www.ncbi.nlm.nih.gov/entrez/query.fcgi?cmd=Retrieve&db=PubMed&dopt=Citation&list_uids=12015615 10.1038/417343a 12015615 PMC6494453

[27] Selverston AI. Invertebrate central pattern generator circuits. Philos Trans R Soc Lond B Biol Sci. 2010;365: 2329–2345. Available from: http://www.hubmed.org/display.cgi?uids=20603355 10.1098/rstb.2009.0270 20603355 PMC2894947

[28] Norris BJ, Coleman MJ, Nusbaum MP. Pyloric motor pattern modification by a newly identified projection neuron in the crab stomatogastric nervous system. J Neurophysiol. 1996;75: 97–108. 10.1152/jn.1996.75.1.97 8822544

[29] Yeh S-Y, Huang W, Wang W, Ward CS, Chao ES, Wu Z, et al Respiratory Network Stability and Modulatory Response to Substance P Require Nalcn. Neuron. 2017;94: 294–303.e4. 10.1016/j.neuron.2017.03.024 28392070 PMC5702257

[30] Daghfous G, Green WW, Alford ST, Zielinski BS, Dubuc R. Sensory Activation of Command Cells for Locomotion and Modulatory Mechanisms: Lessons from Lampreys. Front Neural Circuits. 2016;10: 1–17. 10.3389/fncir.2016.00001 27047342 PMC4801879

[31] Nusbaum MP, Blitz DM. Neuropeptide modulation of microcircuits. Curr Opin Neurobiol. 2012/02/07 ed. 2012;22: 592–601. 10.1016/j.conb.2012.01.003 22305485 PMC3346881

[32] Kiehn O. Locomotor circuits in the mammalian spinal cord. Annu Rev Neurosci. 2006;29: 279–306. 10.1146/annurev.neuro.29.051605.112910 16776587

[33] Le Ray D, Juvin L, Ryczko D, Dubuc R. Supraspinal control of locomotion. The mesencephalic locomotor region. Prog Brain Res. 2011;188: 51–70. 10.1016/B978-0-444-53825-3.00009-7 21333802

[34] Sen R, Wu M, Branson K, Robie A, Rubin GM, Dickson BJ. Moonwalker Descending Neurons Mediate Visually Evoked Retreat in Drosophila. Curr Biol. 2017;27: 766–771. 10.1016/j.cub.2017.02.008 28238656

[35] Schnell B, Ros IG, Dickinson MH. A Descending Neuron Correlated with the Rapid Steering Maneuvers of Flying Drosophila. Curr Biol. 2017;27: 1200–1205. 10.1016/j.cub.2017.03.004 28392112 PMC6309624

[36] Kozlov AK, Kardamakis A a, Hellgren Kotaleski J, Grillner S. Gating of steering signals through phasic modulation of reticulospinal neurons during locomotion. Proc Natl Acad Sci. 2014;111: 3591–3596. 10.1073/pnas.1401459111 24550483 PMC3948313

[37] Morgan PT, Jing J, Vilim FS, Weiss KR. Interneuronal and peptidergic control of motor pattern switching in Aplysia. J Neurophysiol. 2002;87: 49–61. Available from: http://www.ncbi.nlm.nih.gov/entrez/query.fcgi?cmd=Retrieve&db=PubMed&dopt=Citation&list_uids=11784729 10.1152/jn.00438.2001 11784729

[38] Follmann R, Goldsmith CJ, Stein W. Spatial distribution of intermingling pools of projection neurons with distinct targets: A 3D analysis of the commissural ganglia in Cancer borealis. J Comp Neurol. 2017;525: 1827–1843. 10.1002/cne.24161 28001296

[39] Blitz DM, Beenhakker MP, Nusbaum MP. Different sensory systems share projection neurons but elicit distinct motor patterns. J Neurosci. 2004;24: 11381–90. 10.1523/JNEUROSCI.3219-04.2004 15601944 PMC6494448

[40] Hedrich UB, Smarandache CR, Stein W. Differential activation of projection neurons by two sensory pathways contributes to motor pattern selection. J Neurophysiol. 2009;102: 2866–2879. Available from: http://www.ncbi.nlm.nih.gov/entrez/query.fcgi?cmd=Retrieve&db=PubMed&dopt=Citation&list_uids=19741101 10.1152/jn.00618.2009 19741101

[41] Stein W. Modulation of stomatogastric rhythms. J Comp Physiol A Neuroethol Sens Neural Behav Physiol. 2009;195: 989–1009. 10.1007/s00359-009-0483-y 19823843

[42] Hedrich UBS, Stein W. Characterization of a descending pathway: activation and effects on motor patterns in the brachyuran crustacean stomatogastric nervous system. J Exp Biol. 2008;211: 2624–37. 10.1242/jeb.019711 18689416

[43] Beenhakker MP, Blitz DM, Nusbaum MP. Long-lasting activation of rhythmic neuronal activity by a novel mechanosensory system in the crustacean stomatogastric nervous system. J Neurophysiol. 2004;91: 78–91. 10.1152/jn.00741.2003 14523066 PMC6494456

[44] Beenhakker MP, Nusbaum MP. Mechanosensory activation of a motor circuit by coactivation of two projection neurons. J Neurosci. 2004;24: 6741–50. 10.1523/JNEUROSCI.1682-04.2004 15282277 PMC6494447

[45] Nusbaum MP, Weimann JM, Golowasch J, Marder E. Presynaptic control of modulatory fibers by their neural network targets. J Neurosci. 1992;12: 2706–2714. Available from: http://www.ncbi.nlm.nih.gov/entrez/query.fcgi?cmd=Retrieve&db=PubMed&dopt=Citation&list_uids=1613553 1613553 10.1523/JNEUROSCI.12-07-02706.1992 PMC6575831

[46] Blitz DM, Christie AE, Coleman MJ, Norris BJ, Marder E, Nusbaum MP. Different proctolin neurons elicit distinct motor patterns from a multifunctional neuronal network. J Neurosci. 1999;19: 5449–5463. Available from: http://www.ncbi.nlm.nih.gov/entrez/query.fcgi?cmd=Retrieve&db=PubMed&dopt=Citation&list_uids=10377354 10377354 10.1523/JNEUROSCI.19-13-05449.1999 PMC6782314

[47] Goldsmith CJ, Stadele C, Stein W. Optical imaging of neuronal activity and visualization of fine neural structures in non-desheathed nervous systems. PLoS ONE. 2014; 9(7): e103459 10.1371/journal.pone.0103459 25062029 PMC4111610

[48] Städele C, Andras P, Stein W. Simultaneous measurement of membrane potential changes in multiple pattern generating neurons using voltage sensitive dye imaging. J Neurosci Methods. 2011/10/04 ed. 2012;203: 78–88. doi:S0165-0270(11)00563-2 [pii] 10.1016/j.jneumeth.2011.09.015 21963367

[49] Kaiser M. A tutorial in connectome analysis: Topological and spatial features of brain networks. Neuroimage. Academic Press; 2011;57: 892–907. 10.1016/J.NEUROIMAGE.2011.05.025 21605688

[50] Towlson EK, Vertes PE, Ahnert SE, Schafer WR, Bullmore ET. The Rich Club of the C. elegans Neuronal Connectome. J Neurosci. Society for Neuroscience; 2013;33: 6380–6387. 10.1523/JNEUROSCI.3784-12.2013 23575836 PMC4104292

[51] Markov NT, Ercsey-Ravasz M, Van Essen DC, Knoblauch K, Toroczkai Z, Kennedy H. Cortical High-Density Counterstream Architectures. Science (80-). American Association for the Advancement of Science; 2013;342: 1238406–1238406. 10.1126/science.1238406 24179228 PMC3905047

[52] Achard S, Bullmore E. Efficiency and Cost of Economical Brain Functional Networks. PLoS Comput Biol. 2007; 3(2): e17 10.1371/journal.pcbi.0030017 17274684 PMC1794324

[53] Latora V, Marchiori M. Efficient Behavior of Small-World Networks Edited by Foxit Reader. Phys Rev Lett. 2001;87: 3–6. 10.1103/PhysRevLett.87.198701 11690461

[54] Combes D, Meyrand P, Simmers J. Dynamic restructuring of a rhythmic motor program by a single mechanoreceptor neuron in lobster. J Neurosci. 1999;19: 3620–3628. Available from: http://www.ncbi.nlm.nih.gov/entrez/query.fcgi?cmd=Retrieve&db=PubMed&dopt=Citation&list_uids=10212320 10212320 10.1523/JNEUROSCI.19-09-03620.1999 PMC6782242

[55] Venezia JH, Vaden KI, Rong F, Maddox D, Saberi K, Hickok G. Auditory, Visual and Audiovisual Speech Processing Streams in Superior Temporal Sulcus. Front Hum Neurosci. Frontiers Media SA; 2017;11: 174 10.3389/fnhum.2017.00174 28439236 PMC5383672

[56] Petersen CC, Sakmann B. Functionally independent columns of rat somatosensory barrel cortex revealed with voltage-sensitive dye imaging. J Neurosci. 2001/10/19 ed. 2001;21: 8435–8446. Available from: http://www.ncbi.nlm.nih.gov/pubmed/11606632 11606632 10.1523/JNEUROSCI.21-21-08435.2001 PMC6762780

[57] Benedek G, Eördegh G, Chadaide Z, Nagy A. Distributed population coding of multisensory spatial information in the associative cortex. Eur J Neurosci. 2004;20: 525–529. 10.1111/j.1460-9568.2004.03496.x 15233761

[58] Bruno AM, Frost WN, Humphries MD. Modular deconstruction reveals the dynamical and physical building blocks of a locomotion motor program. Neuron. 2015;86: 304–318. 10.1016/j.neuron.2015.03.005 25819612 PMC6016739

[59] Hill ES, Vasireddi SK, Wang J, Bruno AM, Frost WN. Memory Formation in Tritonia via Recruitment of Variably Committed Neurons. Curr Biol. 2015;25: 2879–2888. 10.1016/j.cub.2015.09.033 26549261 PMC4654661

[60] Briggman KL, Abarbanel HD, Kristan WB Jr. Optical imaging of neuronal populations during decision-making. Science (80-). 2005/02/12 ed. 2005;307: 896–901. 10.1126/science.1103736 15705844

[61] Briggman KL, Kristan WB Jr.. Imaging dedicated and multifunctional neural circuits generating distinct behaviors. J Neurosci. 2006/10/20 ed. 2006;26: 10925–10933. 10.1523/JNEUROSCI.3265-06.2006 17050731 PMC6674766

[62] Hartline DK, Maynard DM. Motor patterns in the stomatogastric ganglion of the lobster Panulirus argus. J Exp Biol. 1975;62: 405–420. Available from: http://www.ncbi.nlm.nih.gov/entrez/query.fcgi?cmd=Retrieve&db=PubMed&dopt=Citation&list_uids=173787 173787 10.1242/jeb.62.2.405

[63] Bidaut M. Pharmacological dissection of pyloric network of the lobster stomatogastric ganglion using picrotoxin. J Neurophysiol. 1980;44: 1089–1101. 10.1152/jn.1980.44.6.1089 6256507

[64] Selverston AI, Miller JP, Wadepuhl M. Local circuits for the generation of rhythmic motor patterns. J Physiol. 1982;78: 748–754. 7187449

[65] Christie AE, Stein W, Quinlan JE, Beenhakker MP, Marder E, Nusbaum MP. Actions of a histaminergic/peptidergic projection neuron on rhythmic motor patterns in the stomatogastric nervous system of the crab Cancer borealis. J Comp Neurol. 2004;469: 153–169. Available from: http://www.ncbi.nlm.nih.gov/entrez/query.fcgi?cmd=Retrieve&db=PubMed&dopt=Citation&list_uids=14694531 10.1002/cne.11003 14694531 PMC6494454

[66] Böhm H, Dybek E, Heinzel HG. Anatomy and in vivo activity of neurons connecting the crustacean stomatogastric nervous system to the brain. J Comp Physiol A Sens Neural Behav Physiol. 2001;187: 393–403. Available from: http://www.ncbi.nlm.nih.gov/entrez/query.fcgi?cmd=Retrieve&db=PubMed&dopt=Citation&list_uids=11529483 10.1007/s003590100212 11529483

[67] Clemens S, Massabuau JC, Legeay A, Meyrand P, Simmers J. In vivo modulation of interacting central pattern generators in lobster stomatogastric ganglion: influence of feeding and partial pressure of oxygen. J Neurosci. 1998;18: 2788–2799. Available from: http://www.ncbi.nlm.nih.gov/entrez/query.fcgi?cmd=Retrieve&db=PubMed&dopt=Citation&list_uids=9502835 9502835 10.1523/JNEUROSCI.18-07-02788.1998 PMC6793085

[68] Clemens S, Meyrand P, Simmers J. Feeding-induced changes in temporal patterning of muscle activity in the lobster stomatogastric system. Neurosci Lett. 1998;254: 65–68. Available from: http://www.ncbi.nlm.nih.gov/entrez/query.fcgi?cmd=Retrieve&db=PubMed&dopt=Citation&list_uids=9779921 9779921 10.1016/s0304-3940(98)00511-4

[69] Meijer GT, Montijn JS, Pennartz CMA, Lansink CS. Audio-visual modulation in mouse V1 depends on cross-modal stimulus configuration and congruency. J Neurosci. 2017;37: 0468–17. 10.1523/JNEUROSCI.0468-17.2017 28821672 PMC6596670

[70] Noel J-P, Wallace MT, Orchard-Mills E, Alais D, Van der Burg E. True and Perceived Synchrony are Preferentially Associated With Particular Sensory Pairings. Sci Rep. 2015;5: 17467 10.1038/srep17467 26621493 PMC4664927

[71] Diehl F, White RS, Stein W, Nusbaum MP. Motor circuit-specific burst patterns drive different muscle and behavior patterns. J Neurosci. 2013;33: 12013–12029. 10.1523/JNEUROSCI.1060-13.2013 23864688 PMC3713734

[72] Stein W. Stomatogastric Nervous System [Internet]. Comp. Insect Physiol. Biochem. Pharmacol. Oxford University Press; 2017 10.1093/acrefore/9780190264086.013.153

[73] Sachse S, Galizia CG. The coding of odour-intensity in the honeybee antennal lobe: local computation optimizes odour representation. Eur J Neurosci. 2003/11/19 ed. 2003;18: 2119–2132. Available from: http://www.ncbi.nlm.nih.gov/pubmed/14622173 14622173 10.1046/j.1460-9568.2003.02931.x

[74] Hallock RM, Di Lorenzo PM. Temporal coding in the gustatory system. Neurosci Biobehav Rev. 2006;30: 1145–1160. 10.1016/j.neubiorev.2006.07.005 16979239

[75] Proekt A, Jing J, Weiss KR. Multiple contributions of an input-representing neuron to the dynamics of the aplysia feeding network. J Neurophysiol. 2007;97: 3046–3056. Available from: http://www.ncbi.nlm.nih.gov/entrez/query.fcgi?cmd=Retrieve&db=PubMed&dopt=Citation&list_uids=17314236 10.1152/jn.01301.2006 17314236

[76] Birmingham JT, Szuts ZB, Abbott LF, Marder E. Encoding of muscle movement on two time scales by a sensory neuron that switches between spiking and bursting modes. J Neurophysiol. 1999;82: 2786–2797. Available from: http://www.ncbi.nlm.nih.gov/entrez/query.fcgi?cmd=Retrieve&db=PubMed&dopt=Citation&list_uids=10561445 10.1152/jn.1999.82.5.2786 10561445

[77] Bieler M, Sieben K, Cichon N, Schildt S, Röder B, Hanganu-Opatz IL. Rate and Temporal Coding Convey Multisensory Information in Primary Sensory Cortices. eNeuro. Society for Neuroscience; 2017;4 10.1523/ENEURO.0037-17.2017 28374008 PMC5362936

[78] Nelson AB, Faulstich M, Moghadam S, Onori K, Meredith A, du Lac S. BK Channels Are Required for Multisensory Plasticity in the Oculomotor System. Neuron. Elsevier; 2017;93: 211–220. 10.1016/j.neuron.2016.11.019 27989457 PMC5575767

[79] VanGilder P, Shi Y, Apker G, Dyson K, Buneo CA. Multisensory Interactions Influence Neuronal Spike Train Dynamics in the Posterior Parietal Cortex. PLoS ONE. 2016; 11(12): e0166786 10.1371/journal.pone.0166786 28033334 PMC5199055

[80] Meredith MA, Nemitz JW, Stein BE. Determinants of multisensory integration in superior colliculus neurons. I. Temporal factors. J Neurosci. 1987;7: 3215–3229. Available from: http://www.ncbi.nlm.nih.gov/pubmed/3668625 3668625 10.1523/JNEUROSCI.07-10-03215.1987 PMC6569162

[81] Miller RL, Stein BE, Rowland BA. Multisensory integration uses a real time unisensory-multisensory transform. J Neurosci. 2017;37: 2767–16. 10.1523/JNEUROSCI.2767-16.2017 28450539 PMC5444199

[82] Escanilla OD, Victor JD, Di Lorenzo PM. Odor-taste convergence in the nucleus of the solitary tract of the awake freely licking rat. J Neurosci. Society for Neuroscience; 2015;35: 6284–6297. 10.1523/JNEUROSCI.3526-14.2015 25904782 PMC4405550

[83] Palmer SM, Rosa MG. A distinct anatomical network of cortical areas for analysis of motion in far peripheral vision. Eur J Neurosci. 2006/10/18 ed. 2006;24: 2389–2405. 10.1111/j.1460-9568.2006.05113.x 17042793

[84] Stengl M, Zufall F, Hatt H, Hildebrand JG. Olfactory receptor neurons from antennae of developing male Manduca sexta respond to components of the species-specific sex pheromone in vitro. J Neurosci. 1992;12: 2523–31. 1377232 10.1523/JNEUROSCI.12-07-02523.1992 PMC6575848

[85] Burnett LR, Stein BE, Chaponis D, Wallace MT. Superior colliculus lesions preferentially disrupt multisensory orientation. Neuroscience. 2004;124: 535–547. 10.1016/j.neuroscience.2003.12.026 14980725

[86] Blitz DM, Nusbaum MP. Modulation of circuit feedback specifies motor circuit output. J Neurosci. 2012;32: 9182–9193. Available from: http://www.ncbi.nlm.nih.gov/entrez/query.fcgi?cmd=Retrieve&db=PubMed&dopt=Citation&list_uids=22764227 10.1523/JNEUROSCI.1461-12.2012 22764227 PMC3398468

[87] Nusbaum MP, Blitz DM, Swensen AM, Wood D, Marder E. The roles of co-transmission in neural network modulation. Trends Neurosci. 2001;24: 146–154. Available from: http://www.ncbi.nlm.nih.gov/entrez/query.fcgi?cmd=Retrieve&db=PubMed&dopt=Citation&list_uids=11182454 11182454 10.1016/s0166-2236(00)01723-9

[88] Blitz DM, Nusbaum MP. Neural circuit flexibility in a small sensorimotor system. Curr Opin Neurobiol. 2011;21: 544–552. Available from: http://www.ncbi.nlm.nih.gov/pubmed/21689926 10.1016/j.conb.2011.05.019 21689926 PMC3177960

[89] Blitz DM, Nusbaum MP. State-dependent presynaptic inhibition regulates central pattern generator feedback to descending inputs. J Neurosci. 2008;28: 9564–9574. Available from: http://www.ncbi.nlm.nih.gov/entrez/query.fcgi?cmd=Retrieve&db=PubMed&dopt=Citation&list_uids=18799688 10.1523/JNEUROSCI.3011-08.2008 18799688 PMC2610852

[90] Kirby MS, Nusbaum MP. Central nervous system projections to and from the commissural ganglion of the crab Cancer borealis. Cell Tissue Res. 2007;328: 625–637. Available from: http://www.ncbi.nlm.nih.gov/entrez/query.fcgi?cmd=Retrieve&db=PubMed&dopt=Citation&list_uids=17347812 10.1007/s00441-007-0398-2 17347812

[91] Powers a. R, Hevey MA, Wallace MT. Neural Correlates of Multisensory Perceptual Learning. J Neurosci. 2012;32: 6263–6274. 10.1523/JNEUROSCI.6138-11.2012 22553032 PMC3366559

[92] McGovern DP, Roudaia E, Newell FN, Roach NW. Perceptual learning shapes multisensory causal inference via two distinct mechanisms. Sci Rep. 2016;6: 24673 10.1038/srep24673 27091411 PMC4835789

[93] Duistermars BJ, Frye MA. Multisensory integration for odor tracking by flying Drosophila: behavior, circuits and speculation. Commun Integr Biol. Taylor & Francis; 2010;3: 60–63. 20539786 10.4161/cib.3.1.10076 PMC2881244

[94] Gingras G, Rowland BA, Stein BE. The differing impact of multisensory and unisensory integration on behavior. J Neurosci. Soc Neuroscience; 2009;29: 4897–4902. 10.1523/JNEUROSCI.4120-08.2009 PMC2678542 19369558

[95] Roth E, Hall RW, Daniel TL, Sponberg S. Integration of parallel mechanosensory and visual pathways resolved through sensory conflict. Proc Natl Acad Sci. 2016;113: 12832–12837. 10.1073/pnas.1522419113 27791056 PMC5111652

[96] Yuste R, MacLean JN, Smith J, Lansner A. The cortex as a central pattern generator. Nat Rev Neurosci. 2005;6: 477–483. Available from: http://www.ncbi.nlm.nih.gov/entrez/query.fcgi?cmd=Retrieve&db=PubMed&dopt=Citation&list_uids=15928717 10.1038/nrn1686 15928717

[97] Blitz DM, Nusbaum MP. Motor pattern selection via inhibition of parallel pathways. J Neurosci. 1997/07/01 ed. 1997;17: 4965–4975. Available from: http://www.ncbi.nlm.nih.gov/pubmed/9185534 9185534 10.1523/JNEUROSCI.17-13-04965.1997 PMC6573306

[98] Stein W, Eberle CC, Hedrich UBS. Motor pattern selection by nitric oxide in the stomatogastric nervous system of the crab. Eur J Neurosci. 2005;21: 2767–2781. Available from: http://www.ncbi.nlm.nih.gov/entrez/query.fcgi?cmd=Retrieve&db=PubMed&dopt=Citation&list_uids=15926924 10.1111/j.1460-9568.2005.04117.x 15926924

[99] Obaid AL, Loew LM, Wuskell JP, Salzberg BM. Novel naphthylstyryl-pyridium potentiometric dyes offer advantages for neural network analysis. J Neurosci Methods. 2004/03/09 ed. 2004;134: 179–190. 10.1016/j.jneumeth.2003.11.011 15003384

[100] Quiroga RQ, Nadasdy Z, Ben-Shaul Y. Unsupervised Spike Detection and Sorting with Wavelets and Superparamagnetic Clustering. Neural Comput. 2004;16: 1661–1687. 10.1162/089976604774201631 15228749

[101] Bounova G, De Weck O. Overview of metrics and their correlation patterns for multiple-metric topology analysis on heterogeneous graph ensembles. Phys Rev E—Stat Nonlinear, Soft Matter Phys. American Physical Society; 2012;85: 016117 10.1103/PhysRevE.85.016117 22400635

[102] Bullmore E, Sporns O. Complex brain networks: graph theoretical analysis of structural and functional systems. Nat Rev Neurosci. Nature Publishing Group; 2009;10: 186–198. 10.1038/nrn2575 19190637

