# Supplementary figures and images for: Multimodal sensory information is represented by a combinatorial code in a sensorimotor system
Source: PLoS Biol. 2018 Oct 15;16(10):e2004527. doi: 10.1371/journal.pbio.2004527 (PMC6201955; doi:10.1371/journal.pbio.2004527)

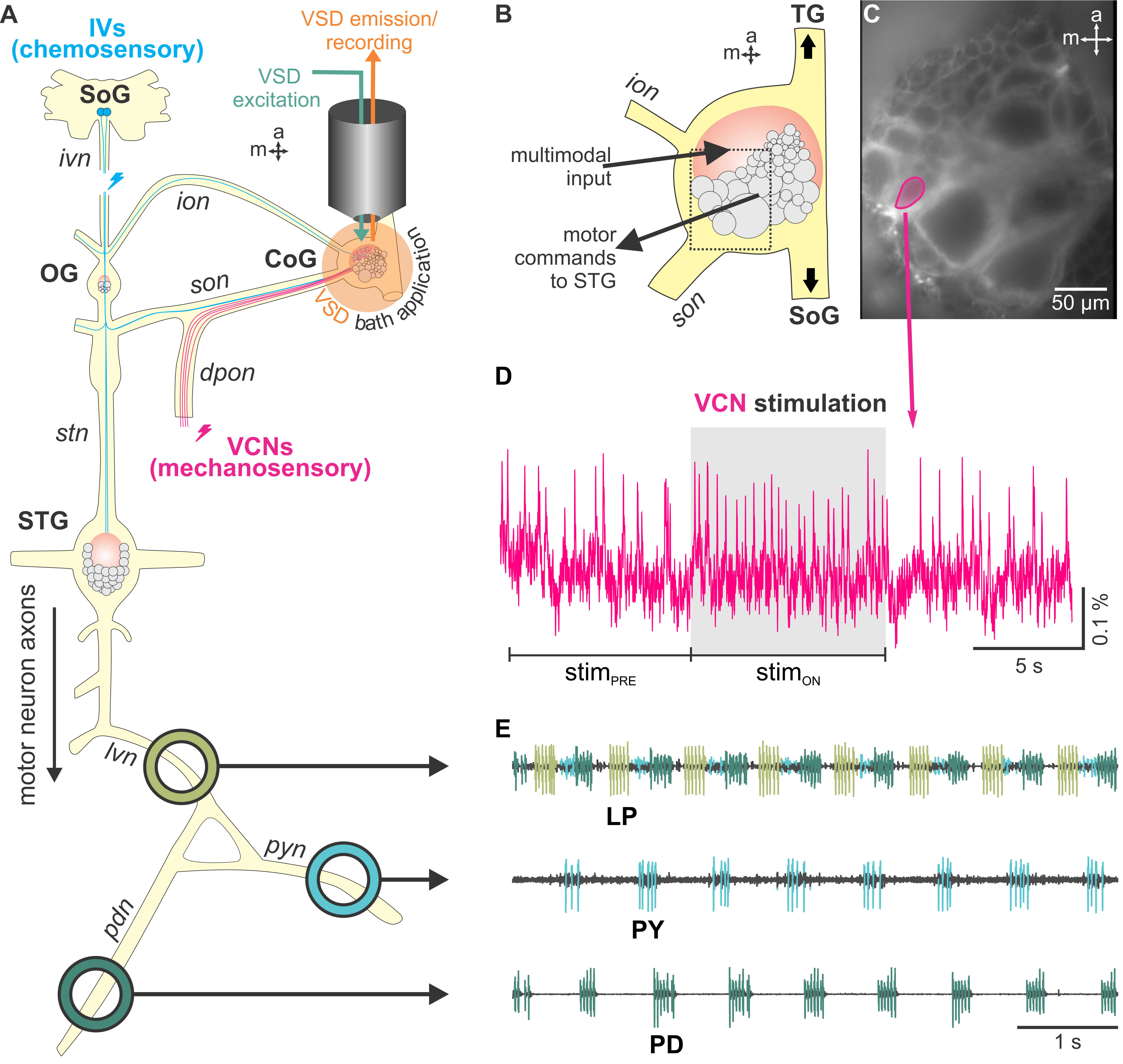

Supplement: S1 Fig — (A) The stomatogastric nervous system consists of four ganglia: the STG, the OG, and the bilaterally paired CoGs (only right CoG shown here). The chemosensory IVs (cyan) descend from the SoG (“brain”) and innervate the CoGs via the ivn, ion, and son and the STG via the stn. The mechanosensory VCNs (magenta) innervate the CoGs via the dpon. VSD imaging was used to monitor CoG neuronal activity. (B) While CoG neurons project to many different locations throughout the nervous system such as the TG and the brain (SoG), a subset of the CoG neurons localized in the medial-posterior area of the ganglion (dotted box) project to downstream motor circuits (arrow) in the STG. (C) Example image of neuronal cell bodies in the CoG area that contains descending projection neurons that control the STG motor circuits. About 80 distinct cell bodies are distinguishable via their brightly fluorescing cell boundaries. All neurons in a single focal plane were imaged simultaneously. (D) Optical trace from a single neuron in (C), showing the change in spike activity in response to VCN stimulation. (E) Sample traces of the downstream pyloric motor neurons recorded extracellularly from motor nerves posterior to the STG. Action potential (spike) information was attained for the LP neuron, the PY neurons, and the PD neurons via recordings of the lvn, the pyn, and the pdn, respectively. CoG, commissural ganglion; dpon, dorsal posterior esophageal nerve; ion, inferior esophageal nerve; IV, inferior ventricular neuron; ivn, inferior ventricular nerve; LP, lateral pyloric; lvn, lateral ventricular nerve; PD, pyloric dilator; pdn, pyloric dilator nerve; PY, pyloric constrictor; pyn, pyloric constrictor nerve; OG, esophageal ganglion; SoG, supraesophageal ganglion; son, superior esophageal nerve; STG, stomatogastric ganglion; stn, stomatogastric nerve; TG, thoracic ganglion; VCN, ventricular cardiac neuron; VSD, voltage-sensitive dye. (TIF) [file pbio.2004527.s001.tif]

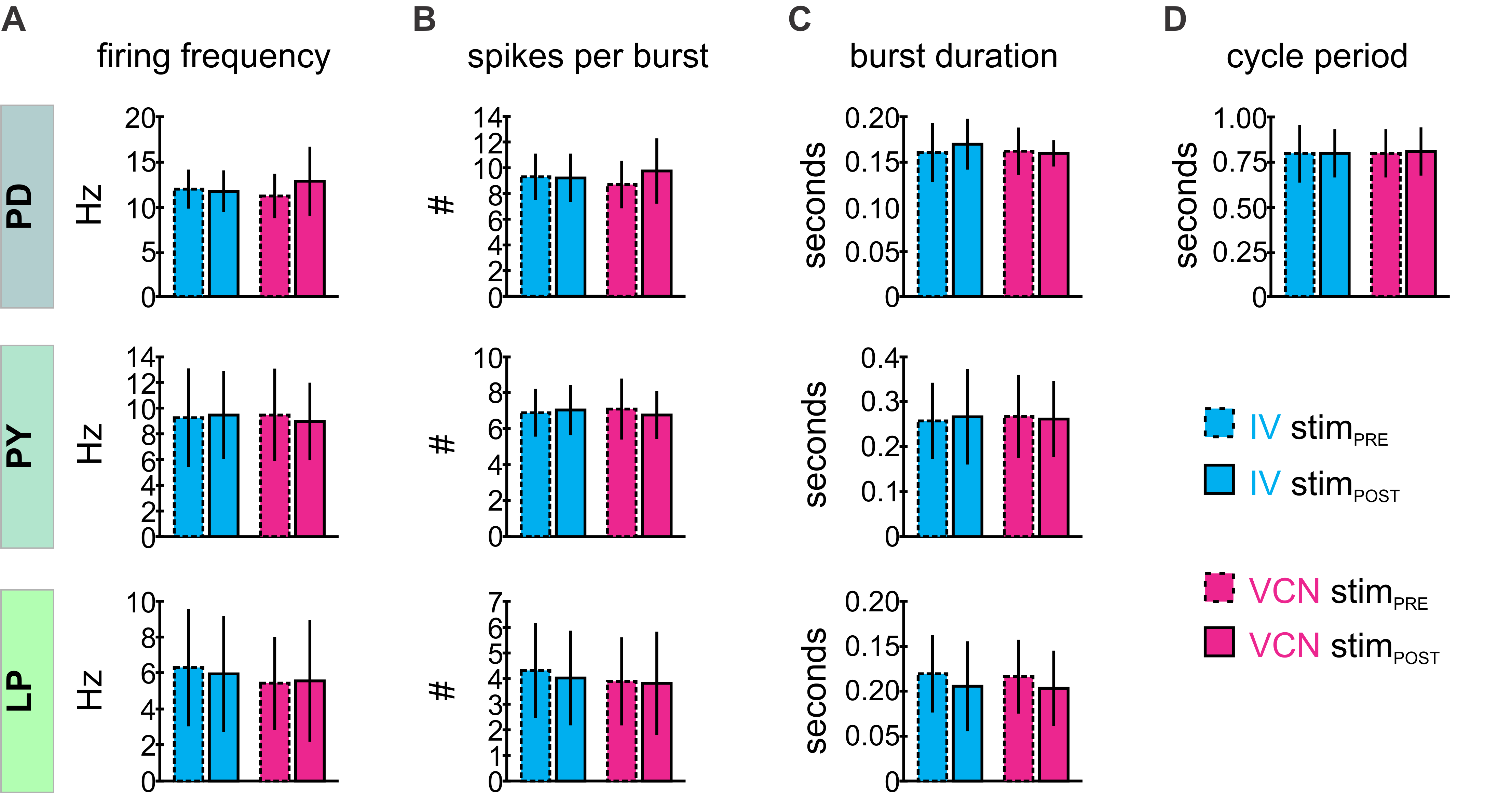

Supplement: S2 Fig — (A-D) Quantification of pyloric rhythm activity before (dashed bars, stimPRE) and after (solid bars, stimPOST) IV stimulation (cyan bars) and VCN stimulation (magenta bars) for PD (top plots), PY (middle plots), and LP (bottom plots). Neuronal firing frequency (A), number of spikes per burst (B), and burst duration (C) were calculated separately for each neuron, while cycle period (D) is a measurement reflective of the whole rhythm. Data are mean ± SD. Comparisons were made within modalities, and no differences were found between stimPRE and stimPOST activity (paired t test; no significance; N = 8 ganglia, 5 crabs, for PD; N = 7 ganglia, 4 crabs, for PY; and N = 7 ganglia, 6 crabs, for LP). CoG, commissural ganglion; IV, inferior ventricular neuron; LP, lateral pyloric; PD, pyloric dilator; PY, pyloric constrictor; STG, stomatogastric ganglion; VCN, ventricular cardiac neuron. (TIF) [file pbio.2004527.s002.tif]
